# Comparative insights into the saccharification potentials of a relatively unexplored but robust *Penicillium funiculosum* glycoside hydrolase 7 cellobiohydrolase

**DOI:** 10.1186/s13068-017-0752-x

**Published:** 2017-03-20

**Authors:** Funso Emmanuel Ogunmolu, Navya Bhatt Kammachi Jagadeesha, Rakesh Kumar, Pawan Kumar, Dinesh Gupta, Syed Shams Yazdani

**Affiliations:** 1 Microbial Engineering Group, International Centre for Genetic Engineering and Biotechnology, Aruna Asaf Ali Marg, New Delhi, 110067 India; 2 Translational Bioinformatics Group, International Centre for Genetic Engineering and Biotechnology, New Delhi, India; 3 DBT-ICGEB Centre for Advanced Bioenergy Research, International Centre for Genetic Engineering and Biotechnology, New Delhi, India

**Keywords:** Biomass degradation, Cellobiohydrolase 1, CAZymes: glycoside hydrolases: *Penicillium funiculosum*, Molecular dynamics

## Abstract

**Background:**

GH7 cellobiohydrolases (CBH1) are vital for the breakdown of cellulose. We had previously observed the enzyme as the most dominant protein in the active cellulose-hydrolyzing secretome of the hypercellulolytic ascomycete—*Penicillium funiculosum* (NCIM1228). To understand its contributions to cellulosic biomass saccharification in comparison with GH7 cellobiohydrolase from the industrial workhorse—*Trichoderma reesei*, we natively purified and functionally characterized the only GH7 cellobiohydrolase identified and present in the genome of the fungus.

**Results:**

There were marginal differences observed in the stability of both enzymes, with *P. funiculosum* (PfCBH1) showing an optimal thermal midpoint (*T*_m_) of 68 °C at pH 4.4 as against an optimal *T*_m_ of 65 °C at pH 4.7 for *T. reesei* (TrCBH1). Nevertheless, PfCBH1 had an approximate threefold lower binding affinity (*K*_m_), an 18-fold higher turnover rate (*k*_cat_), a sixfold higher catalytic efficiency as well as a 26-fold higher enzyme-inhibitor complex equilibrium dissociation constant (*K*_i_) than TrCBH1 on *p*-nitrophenyl-β-d-lactopyranoside (*p*NPL). Although both enzymes hydrolyzed cellooligomers (G2–G6) and microcrystalline cellulose, releasing cellobiose and glucose as the major products, the propensity was more with PfCBH1. We equally observed this trend during the hydrolysis of pretreated wheat straws in tandem with other core cellulases under the same conditions. Molecular dynamic simulations conducted on a homology model built using the TrCBH1 structure (PDB ID: 8CEL) as a template enabled us to directly examine the effects of substrate and products on the protein dynamics. While the catalytic triads—EXDXXE motifs—were conserved between the two enzymes, subtle variations in regions enclosing the catalytic path were observed, and relations to functionality highlighted.

**Conclusion:**

To the best of our knowledge, this is the first report about a comprehensive and comparative description of CBH1 from hypercellulolytic ascomycete—*P. funiculosum* NCIM1228, against the backdrop of the same enzyme from the industrial workhorse—*T. reesei*. Our study reveals PfCBH1 as a viable alternative for CBH1 from *T. reesei* in industrial cellulase cocktails.

**Electronic supplementary material:**

The online version of this article (doi:10.1186/s13068-017-0752-x) contains supplementary material, which is available to authorized users.

## Background

Cellobiohydrolases (CBHs, cellulose 1,4-β-cellobiosidases, EC 3.2.1.91) of the glycoside hydrolase family 7 are among the most important cellulolytic enzymes both in nature and for emerging industrial applications for crystalline cellulose breakdown [1–3]. They are mainly found in eukaryotes, of which reports of discoveries in filamentous fungi predominates, and are among the most common cellulolytic enzymes in secretomes of biomass-degrading fungi produced under cellulase-inducing conditions [3–5]. They act from the reducing ends of cellulose chains, latching onto the cellulose substrates and processively release cellobiose until they run into obstructions or are inactivated [6–8].

Like other glycoside hydrolases, they efficiently accelerate the hydrolysis of the glycosidic bonds in the crystalline cellulose by more than 10^17^-fold, making them one of the most efficient catalysts known [7, 9, 10]. The first discovered and best characterized GH7 CBH was from the industrial workhorse *Trichoderma reesei* [1, 3]; since then, GH7 CBH from *T. reesei* has been one of the major components of commercial cellulase cocktails [4, 11, 12]. Albeit, reports of alternatives with higher potentials—higher specific activity, less inhibition to cellobiose and lignin-derived compounds—from genus *Penicillium*, *Humicola*, *Acremonium* among others abound [4].

Most GH7 CBHs from filamentous fungi are multi-modular in nature, consisting of a carbohydrate-binding module (CBM), an *O*-glycosylated linker, and a large catalytic domain (CD) containing a tunnel for threading cellulose chain [13, 14]. The tunnel-bearing structures allow the enzyme to slide along the cellulose chain to the next cleavage site as the product is released [2]. GH7 CBHs catalytic domain shares a common β-jelly roll fold with two largely antiparallel β-sheets packing face to face to form a curved β-sandwich. Long loops extend the edges of the β-sandwich and form a long substrate-binding groove along the entire GH7 catalytic module. [3, 14–17].

The majority of reported differences, however, were observed with length and sequence of loops around substrate-binding paths, catalytic centers or products binding sites [3, 14–16, 18]. Till date, GH7 CBH from *Trichoderma reesei* (TrCBH1) exhibits the most extensively enclosed tunnel among known GH7 CBH structures, while *Phanerochaete chrysosporium* Cel7D (PchCel7D) displays the most open active site due to several loop deletions and residue size reductions on the tips of tunnel-enclosing loops [15]. These loop variations gave a more accessible active site and had been adduced as partly responsible for PchCel7D’s enhanced activity on small soluble substrates, as well as tolerance to cellobiose inhibition [3, 15, 18].

*Penicillium funiculosum* (NCIM1228) on the other hand is a filamentous fungus isolated from the Indian subcontinent. Our previous work on the strain identified it as a hypercellulolytic fungus. We also discovered that it has only one gene coding for GH7 cellobiohydrolase (PfCBH1) and that the enzyme is possibly the most important protein in cellulose-hydrolyzing secretome based on its abundance and distribution [5]. However, the properties of the enzyme had not been previously explored or reported. To this end, we reported here the purification and functional characterization of the GH7 CBH from *Penicillium funiculosum* (NCIM1228) and compared its performances with that from *Trichoderma reesei* (TrCBH1).

Furthermore, we built a PfCBH1 three-dimensional structure using the TrCBH1 structure as a template and conducted molecular dynamics simulations to compare the structural differences of PfCBH1 and TrCBH1 catalytic domains in solution without a bound ligand, in solution bound to a cellononaose ligand, and in the presence of cellobiose as a product cum inhibitor. Simulations of the enzymes in each representative state enabled us to directly examine the effects of substrate and products on protein dynamics. Understanding the diversity of these key industrial enzymes is critical to engineering them for higher levels of activity and greater stability which will in turn significantly aid in the commercialization of biofuel processes based on enzymatic depolymerization of polysaccharides [3, 14–16, 18].

## Results and discussion

### Biochemical characterisation of PfCBH1

**Fig. 1:**
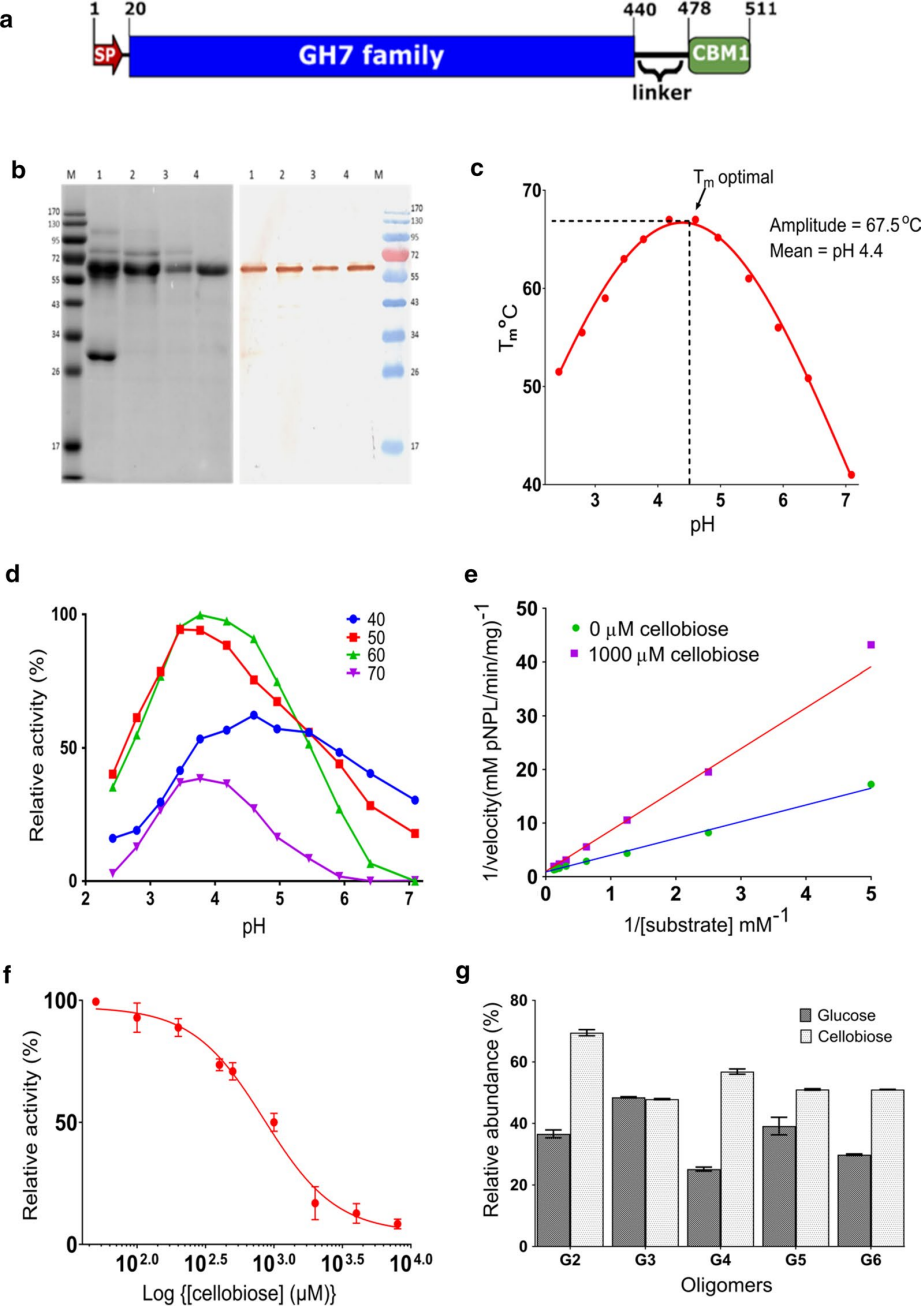
Properties of PfCBH1. **a** The schematic representation of the amino acid sequence encoded by the *PfCBH1* gene. The picture was generated with IBS v1.0 (http://ibs.biocuckoo.org/); signal peptides prediction was made using services of the SignalP 4.1 server (http://www.cbs.dtu.dk/services/SignalP/) and domain prediction with Pfam (http://pfam.xfam.org/). **b** The SDS-PAGE and Western blot confirmation using anti-PfCBH1 polyclonal antibody. Crude enzyme (*lane 1*) from the most performing secretome of *P. funiculosum* was subjected to hydrophobic interaction chromatography (*lane 2*), followed by anion exchange chromatography separation of active fractions (*lane 3*), the flow through was further subjected to hydrophobic interaction chromatography (*lane 4*) yielding pure CBH1 enzyme. M is a protein molecular weight marker. **c** The thermal stability of purified PfCBH1 under different pH conditions. The T_m_ optimal and pH are reported as amplitudes and means of the Gaussian fittings, respectively. **d** The relative Avicelase activity of purified PfCBH1 under different pH and temperature conditions. **e** The Lineweaver–Burk plot revealing the competitive nature of the inhibition by cellobiose. **f** The Log(inhibitor) vs. response curve for IC50 determination. Data are expressed as a percentage of uninhibited activity. A Hill slope of −1.6 was obtained implying a reduction in affinity for *p*NPL in the presence of cellobiose. **g** The hydrolysis of oligosaccharides by PfCBH1. The oligosaccharides tested are cellobiose (*G2*), cellotriose (*G3*), cellotetraose (*G4*), cellopentaose (*G5*), and cellohexaose (*G6*)

The preliminary analysis of PfCBH1 nucleotide and the encoded protein sequence showed that it consists of 1536 bp without introns. It encodes 511 amino acids, categorized into a 17-residue signal peptide, a GH7 catalytic module of ~420 residues, a linker region of ~38 residues, and finally a C-terminal CBM1 of ~33 residues (Fig. 1a).

To characterize the protein and compare its properties with previously characterized cellobiohydrolases of the GH7 family, most especially cellobiohydrolase 1 from the industrial workhorse—*T. reesei* (TrCBH1), we purified PfCBH1 from the crude secretome to homogeneity using a three-step purification method (Additional file 1: Figure S1; Table S1A, B). The purity and the identity of the protein were confirmed by SDS-PAGE and Western blot analysis (Fig. 1b). The detection of the single band on the Western blot corresponding to the approximate ~60 kDa on SDS-PAGE confirms the identity of the purified protein. We observed that the molecular weight of the purified protein was higher in comparison to the molecular mass deduced from the amino acid sequence (53 kDa) possibly due to glycosylation. GH7 CBHs are known to be highly glycosylated, with both *O*-linked and *N*-linked glycosylations [3, 14, 19].

The effect of temperature and pH interdependence on the stability of PfCBH1 and, for comparison, TrCBH1 was determined through differential scanning fluorimetry employing SYPRO Orange. SYPRO Orange is an environmentally sensitive dye. The unfolding process exposes the hydrophobic region of proteins and results in an increase in fluorescence, which is used to monitor the protein unfolding transition [20]. In our experiments, the observed thermal midpoint (*T*_m_) values of the purified enzymes varied considerably under different pH conditions (Fig. 1c). PfCBH1 showed a relatively higher thermostability at acidic pH when compared to TrCBH1 under the same circumstances. PfCBH1 exhibited an optimal *T*_m_ of 68 °C at pH 4.4 as against an optimal *T*_m_ of 65 °C at pH 4.7 for TrCBH1 (Additional file 1: Figure S2).

The interdependence of purified PfCBH1 activity on the reaction temperature and pH was equally evaluated using Avicel as the substrate, and the Avicelase activity was determined using similar conditions for thermal shift assay. The data point with the highest activity (0.16 U/mg) was regarded as the optimum (Fig. 1d). Greater than 75% of Avicelase activity was maintained between 50 and 60 °C and pH range about 3.2–4.6 (Fig. 1d). The obtained values were in good agreement with optimal conditions for fungal cellobiohydrolases 1 [3]. For consistency, we decided to evaluate the kinetics and substrate specificity assays of PfCBH1 at pH 4.4 and temperature 50 °C.

The specific activity of our purified PfCBH1 against microcrystalline cellulose (Avicel PH-101), and chromogenic substrates *p*-nitrophenyl-β-d-cellobioside (*p*NPC) and *p*-nitrophenyl-β-d-lactopyranoside (*p*NPL) are shown in (Table 1). However, the kinetic parameters were estimated on *p*NPL (Table 2). The values obtained were compared with a previously published data for TrCBH1 on *p*NPL [18]. The *K*_m_ value of PfCBH1 was about threefold higher than the reported *K*_m_ value for TrCBH1 indicating a comparative lower binding affinity for *p*NPL. PfCBH1, on the other hand, showed an approximately 18-fold higher turnover rate (*k*_cat_) as well as a sixfold higher catalytic efficiency on *p*NPL than TrCBH1 (Table 2). These parameters were equally evaluated in the presence of 1000 µM cellobiose to understand the mechanism of cellobiose inhibition of PfCBH1 and its tolerance.

**Table 1:** Specific activity of purified cellobiohydrolase 1 (*PfCBH1*) of *P. funiculosum*

Substrates	*p*NPL	*p*NPC	Avicel
Specific activity (U/mg)	0.27 ± 0.003	0.11 ± 0.002	0.14 ± 0.001

**Table 2:** Biochemical and kinetic characterization of *PfCBH1* using *p*NPL as substrate

Parameters	PfCBH1	TrCBH1^a^
*V*_max_ (mM/min ± SE)	0.3 ± 0.1	NR
*K*_m_ (mM ± SE)	3.5 ± 0.2	1.2 ± 0.1
*k*_cat_ (min^−1^ ± SE)	295 ± 7	16 ± 3
*k*_cat_/*K*_m_ (M^−1^ s^−1^)	1421	224
*K*_i_ (µM ± SE)	760 ± 54	29

^a^Values for TrCBH1 were published data obtained from [18]

Consistent with competitive inhibition, the presence of cellobiose resulted in increased *K*_m_ values from 3.5 to 7.4 µM for *p*NPL, whereas the catalytic constant remained unaffected (Fig. 1e). Competitive inhibition is a common trend in cellobiohydrolase 1 families when evaluated on soluble substrates [3, 18, 21]. We obtained an equilibrium dissociation constant of an enzyme-inhibitor complex (*K*_i_) value 26-fold higher than that reported for TrCBH1 (Table 2) [18, 22]. The *K*_i_ being the best parameter for describing the inhibitory strength of an inhibitor is directly related to the thermodynamic stability of the enzyme-inhibitor complex [23]. The hydrolysis of *p*NPL in the presence of increasing concentrations of cellobiose revealed an IC_50_ value of 849 µM (Fig. 1F, Additional file 1: Figure S3).

The product profiles generated upon incubating PfCBH1 with cellooligomers (G2–G6) are shown in Fig. 1g. As expected, PfCBH1 was active on all the substrates except cellobiose, releasing cellobiose and glucose as the major products. Cellobiose is a known product generated from the processive hydrolysis of long chain glucose units linked in the β-1,4-conformations and glucose is released due to initial hydrolysis events [3, 12, 16, 21]. We, however, noticed about 40% glucose released when cellobiose was the substrate. This suggests that PfCBH1 not only tolerate but might possess inherent cellobiose hydrolysis ability. Kern et al. [16] and Texier et al. [22] had earlier on reported similar observations with GH7 cellobiohydrolase from *Limnoria quadripunctata* and *Penicillium funiculosum* IMI 378536, respectively.

### Comparative evaluation of PfCBH1 and TrCBH1 saccharification potentials on polymeric cellulosic substrates

**Fig. 2:**
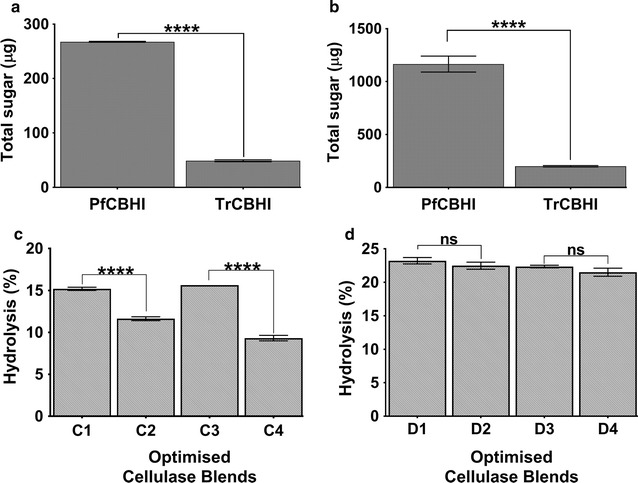
Comparative saccharification potentials of PfCBH1 and TrCBH1 on polymeric cellulosic substrates. **a**, **b** The amount of total sugar obtained from the hydrolysis of microcrystalline cellulose (Avicel) using the purified GH7 CBH’s after 1- and 24-h incubation, respectively, while **c** and **d** show the hydrolysis potentials of optimized blends on ammonium hydroxide and sodium hydroxide pretreated wheat straws, respectively. In **c**, cellulase blend C1 contain—PfCBH1 to TrCBH1 ratio—[39:7], C2 is an inversion with—PfCBH1 to TrCBH1 ratio—[7:39]; C3 contains only PfCBH1 at 46%, while C4 contains only TrCBH1 at 46%. In **d**, cellulase blend D1 contain—PfCBH1 to TrCBH1 ratio [5:34]; D2 is an inversion with-PfCBH1 to TrCBH1 ratio—[34:5]; D3 contains only PfCBH1 at 39%; while D4 contains only TrCBH1 at 39%. All other components were kept as shown in (Additional file 1: Table S3). ****p < 0.0001, while ns: no significant difference at α = 0.05 using Tukey’s multiple comparison test. *Error bars* represent ±SE

Given the observed enzymatic parameters obtained for PfCBH1 suggesting improved kinetic properties when compared with TrCBH1, we at first evaluated the saccharification potentials of the two enzymes singly on microcrystalline cellulose (Avicel). Both enzymes were active on Avicel releasing cellobiose and glucose. PfCBH1, however, released more sugars (Fig. 2a, b).

The abilities of PfCBH1 and TrCBH1 to also bring about the saccharification in tandem with other core cellulases (procured from commercial vendors) on lignocellulosic biomass were equally evaluated using ammonium hydroxide (AMM) and sodium hydroxide (ALK) pretreated wheat straws, respectively. The properties of the tested enzymes are indicated in (Table 3), while the compositional analysis of the differentially pretreated biomass (gifted by Dr. Arvind Lali) in Additional file 1: Table S2. Reports abound on the importance of the various enzyme components being essential for biomass hydrolysis [24, 25]. As such, we set up the experiments to evaluate the saccharification potentials of the two cellobiohydrolases (PfCBH1 and TrCBH1), in synergy with the other core cellulases.

**Table 3:** Properties of core cellulases used in the biomass hydrolysis experiments

	Enzyme	CAZy classification	Source organism	Molecular weight (kDa)	Vendor/source
A	Cellobiohydrolase I (TrCBH1)	GH7	*Trichoderma reesei* (expressed in corn)	55	Sigma, USA
B	β-Glucosidase (BGL)	GH3	*Aspergillus niger*	121	Megazyme, Ireland
C	β-Xylanase (BXYL)	GH11	*Aspergillus niger*	25	Megazyme, Ireland
D	Endo-β-glucanase (ENDO)	GH5	*Talaromyces emersonii*	42, 37	Megazyme, Ireland
E	Endo-β-glucanase (ENDO)	GH7	*Trichoderma* sp.	57	Megazyme, Ireland
F	Cellobiohydrolase I (PfCBH1)	GH7	*Penicillium funiculosum*	55	This study
G	Cellobiohydrolase II (CBHII)	GH6	*Recombinant*	47	Megazyme, Ireland

Our aim was to ascertain the saccharification potentials of the two cellobiohydrolases (PfCBH1 and TrCBH1) in a scenario where product inhibition is mostly eliminated by the presence of the partner enzymes. Payne et al. [3] had earlier on stated that the rate-limiting step in GH7 CBHs actions in the absence of synergistic enzymes is likely to be substrate dissociation, either caused by obstacles or amorphous regions of cellulose. On the other hand, the presence of synergistic enzymes is likely to enhance the processive velocity of GH7 CBHs through the provision of points of detachment, thereby removing the rate limitation of substrate dissociation.

Thus, using the optimal enzyme blends as suggested by Design Expert^®^, we estimated the biomass hydrolysis potentials of PfCBH1 and TrCBH1 in the presence of other core cellulolytic enzymes (Additional file 1: Table S3). The optimal enzyme blends of TrCBH1, BGL, BXYL, ENDO5, ENDO7, PfCBH1, and CBHII were in the ratios 7:5:0:35:10:39:5 for AMM, and 34:5:11:27:4:5:15 for ALK (Additional file 1: Table S4). Our data showed a 15% hydrolysis on AMM pretreated wheat straw (Fig. 2c) and a 23% hydrolysis on ALK pretreated wheat straw under similar conditions (Fig. 2d). We wish to state that the modest percentage hydrolysis obtained in this experiment is attributable to the low enzyme loadings (2.5 mg/g dry matter).

To now ascertain the differential abilities of PfCBH1 and TrCBH1 in bringing about biomass saccharification in synergy with other core cellulases, in one instance, we interchanged the predicted ratios of PfCBH1 and TrCBH1 in the optimal enzyme blends (Additional file 1: Table S4), while keeping the concentration of other enzymes at the predicted values. In another instance, we excluded either of PfCBH1 or TrCBH1 from the enzyme blends while the retained CBH1 fraction assumes the sum of the predicted values for GH7 CBHs in the blends. In the two instances, enzyme blends containing PfCBH1 outperformed blends with TrCBH1 on AMM-treated wheat straws (Fig. 2c), with a 31% reduction in hydrolysis between blends C1 and C2 and a 67% reduction in hydrolysis between blends C3 and C4.

On the other hand, while we observed a 4% reduction in hydrolysis between blends D1 and D2 as well as between blends D3 and D4 on ALK-pretreated wheat straws, the differences were not statistically significant at *p* < 0.05 using Tukey’s multiple comparison test (Fig. 2d). These differential actions we could attribute to the biomass properties occasioned by the pretreatment regimens they have undergone. In this context, the AMM-pretreated biomass seemed more recalcitrant than its ALK counterpart (Additional file 1: Table S2). After all, the type of pretreatment a biomass undergoes affect the outcome of enzyme hydrolysis of such lignocellulosic biomass [25, 26].

The difference in the biomass properties is further highlighted in a follow-up investigation in which we observed that ENDO5 (a GH5 endoglucanase) from *Talaromyces emersonni* released sugars from ALK-pretreated wheat straw at the similar magnitude with PfCBH1 but not on AMM-pretreated wheat straw (Additional file 1: Figure S4).

### Computational evaluation of PfCBH1 and TrCBH1 interactions with cellulose

After an extensive biochemical and functional comparison between PfCBH1 and TrCBH1, we wanted to understand the possible explanations for the observed differences in molecular interactions using computational modeling and simulation methods. The methods hold the prospect of offering insights that are complementary to biochemical experiments for developing a detailed structure–function relationship, cellulase–cellulose interactions and for designing enhanced enzymes for biomass conversion [3, 13–15, 27–33]. However, there are no 3D structures available for PfCBH1 in public repositories, but there were 13 experimentally determined 3-dimensional structures of cellobiohydrolase 1 in the PDB database (as on September 2016). We thus retrieved the full-length amino acid sequences corresponding to these PDB entries and performed a multiple sequence alignment of the sequences with PfCBH1, trimming off regions flanking the GH7 domain (e.g., the signal peptide, CBM, linker) (Additional file 1: Figure S5). The similarities between the sequences are depicted with normalized sequence logo beneath the alignments.

**Fig. 3:**
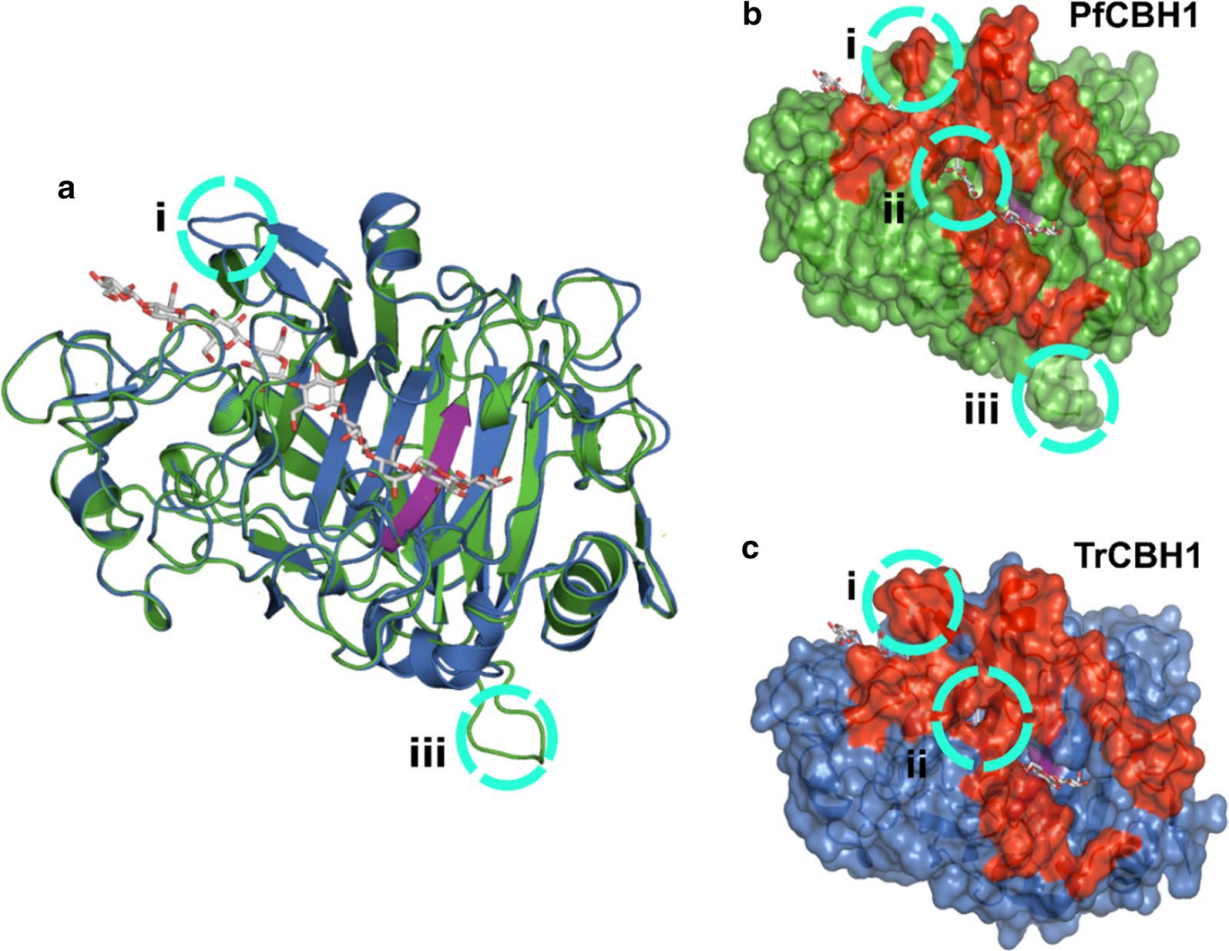
Analysis of PfCBH1 and TrCBH1 models. **a** The superposition of the structures of TrCBH1 and PfCBH1. **b**, **c** The space-filled structures comparing the substrate tunnel enclosures of CBH1 from *P*. *funiculosum* (*green colored*), and *T*. *reesei* (*blue colored*), respectively. The *red-colored* regions correspond to the loops along the substrate-binding path, while the catalytic triad region is *highlighted in purple*. The obviously different regions are* highlighted in dotted circles* and labeled accordingly. In all frames, the cellononaose ligand from the TrCBH1 Michaelis complex is shown as *gray sticks*

Consistent with the maxim that proteins with evolutionary relationships assume a certain percentage of their amino acid residues conserved, we observed a reasonable degree of amino acid conservation between the various catalytic domains retrieved; as well as a moderately high pairwise sequence identity between each of the retrieved proteins and PfCBH1 (Additional file 1: Figure S5). However, because TrCBH1 is the most characterized protein of the CBH1 families, the protein of choice for our biochemical comparison, as well as the GH7 CBH of choice for most industrial cellulase cocktails, we constructed a homology model of PfCBH1 using a TrCBH1 structure as a template (Additional file 1: Figure S6A). The corresponding Φ and Ψ distributions of the non-glycine, non-proline residues on the Ramachandran plot are summarized in Additional file 1: Figure S6B. The superimposition of the obtained PfCBH1 structure with TrCBH1 structure is equally demonstrated in Fig. 3a.

As expected from previous reports [3, 14, 15, 18, 34, 35], the overall folds in the catalytic modules of PfCBH1 and TrCBH1 were very similar to one another with root mean square deviation of 0.12 Å for 339 matching Cα atoms. The cellulose binding sites are highly conserved, including the catalytic triads—EXDXXE motifs—as shown in Additional file 1: Figures S5 and S6. All previously identified loops along the substrate-binding path (A1 to A4 and B1 to B4) were equally observed revealing a highly enclosed active site, characteristic of GH7 cellobiohydrolases. The major differences, however, were highlighted as circles i, ii, and iii, respectively (Fig. 3a–c). Circle i, also corresponding to the consensus loop A1, is present at the binding tunnel entrance, and it is shorter in PfCBH1. A closer look indicates that the shortening is due to the deletion of 3 amino acid residues—S, A, and E—when compared with the corresponding region in TrCBH1 (Additional file 1: Figure S5). The deletion is critical in defining the overall opening profile of the ligand binding site and believed to be responsible for ‘the more open’ architecture of the substrate-binding tunnel entrance [3, 34, 36]. Similar deletions have been reported in GH7 CBH’s from *Talaromyces emersonii* [37], *Trichoderma harzianum* [36], and *Phanerochaete chrysosporium* [34].

On difference “circle iii”—adjoining loop adjacent to loop B4—we observed that the region comprising of eight amino acids (D, G, T, S, T, G, S, and L) is natively missing in the TrCBH1 catalytic module (Additional file 1: Figure S5). There seems to be no direct involvement of this region and the substrate processing in the tunnel. However, the proximity of this loop to loop B4, present at the product side of the active site tunnel [14], could suggest its possible participation in product inhibition alleviation. On the other hand, the open cavity formed at the sidewalls of the ligand binding tunnel (circle ii) has been reported in [34, 36] and found to be responsible for the easier reorientation and thoroughfare of substrates to the catalytic sites (Fig. 3b, c). The possible interactions between adjacent loops B2 and B3 with opposing loop A3 across the active site account for the opening and closing as well as the substrate accessibility to the active site. A closed tunnel suggests that a cellulose chain may only reach the catalytic center by threading from the tunnel entrance, while a more open configuration allows for endo-initiation of cellulose hydrolysis [3, 38]. Also, a higher flexibility along the active site may enhance the rate of enzyme detachment from the cellulose substrate and may also reduce product inhibition, although this comes with a decrease in the degree of processivity as a trade-off [3, 18, 34, 38, 39].

**Fig. 4:**
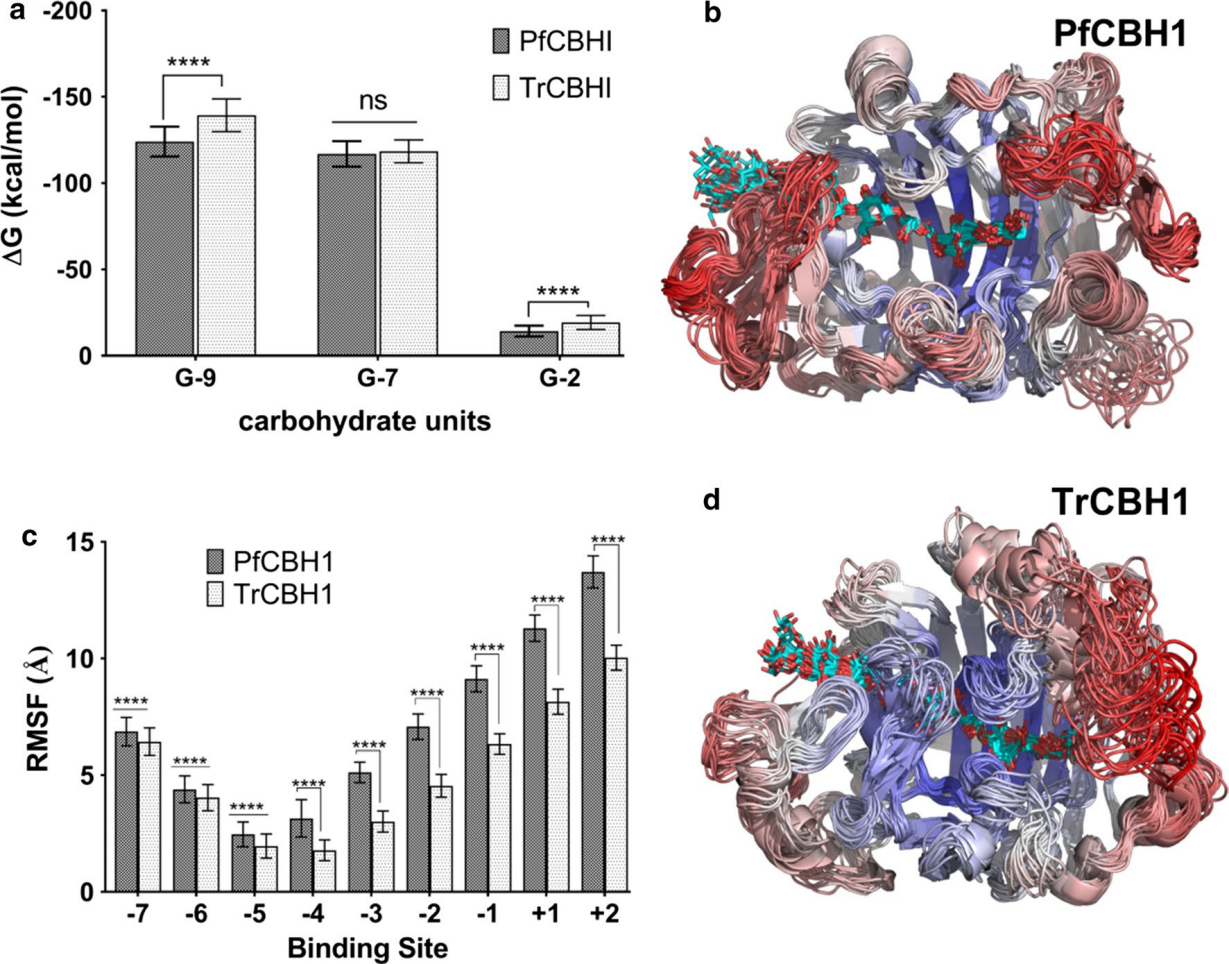
MD simulations of PfCBH1 and TrCBH1 catalytic domains. **a** The energy decomposition comparison between PfCBH1 and TrCBH1 in the presence of cellononaose (*G-9*), celloheptaose (*G-7*), and cellobiose (*G-2*). Binding energies were derived from Molecular Mechanics Generalized Born Surface Area (MMGBSA) calculations. The significance discovery between groups is determined using the Two-stage linear step-up procedure of Benjamini, Krieger, and Yekutieli. ****p < 0.0001, while ns: no significant difference at α = 0.05. **b**, **d** The cluster representations of PfCBH1 and TrCBH1 over a 100-ns trajectory at 5-ns intervals. The enzymes are colored by RMSF, where *red* represents the highest fluctuations, and *blue* represents the lowest fluctuations. **c** The root-mean-square fluctuation (RMSF) of the active site-bound cellononaose by binding subsite. The RMSF values were calculated based on the glucose-heavy atoms over the entire 100-ns MD simulation. The *error bars* were computed by block averaging. ****p < 0.0001, at α = 0.05

To complement the insights offered by static geometry comparison above, we conducted molecular dynamics simulations of PfCBH1 and TrCBH1 catalytic domains in solution without a bound ligand, in solution bound to a cellononaose ligand, and in the presence of celloheptaose plus cellobiose. Simulations of the enzymes in each representative state enabled us to directly examine the effects of substrate—cellononaose and product—cellobiose, on protein dynamics. The energy decomposition of the two proteins under the different simulation environment is shown in Fig. 4a. Longer chain oligomers—cellononaose and celloheptaose were more energetically favored than dimer—cellobiose; this is characteristic of GH7 cellobiohydrolases [2, 3, 6–8, 11, 12, 17, 40, 41]. Of the two proteins under consideration, TrCBH1 seemed to be more energetically favored both on cellononaose as well as cellobiose, while no significant difference was observed on celloheptaose. The implication of this is that TrCBH1 binds more to both cellononaose (confirming lower *K*_m_ observed for TrCBH1) as well as cellobiose. While higher affinity to cellononaose is a desirable attribute for GH7 CBHs, the non-productive binding to cellobiose is a drawback; leading to a higher propensity for inhibition of the enzyme.

For each simulation, the root mean square deviations of the proteins backbones were calculated, and the results are provided in Additional file 1: Figure S7. Our results show that the proteins were mostly stable and reached geometrical convergence after about 20 ns timescale in the presence or absence of a bound ligand as well as the product. The RMSD averaged, in general, an approximate 2 Å units under 100 ns timescale simulations. With the exception of the apo forms of the proteins where TrCBH1 exhibited a better stability than PfCBH1, we observed a relatively stable RMSDs with PfCBH1. This was more pronounced in the presence of celloheptaose plus cellobiose (a mimic of substrate-product complex transition). Variations about 5 Å were observed after about 40 ns in TrCBH1 as against a relatively stable (1 Å) variation in PfCBH1 under the same condition. This could partly explain the differences in cellobiose tolerance by the two proteins. Our previous observation with PfCBH1 is that it tolerates more of cellobiose than TrCBH1 (Table 2; Additional file 1: Figure S3). A better RMSD stability in the presence of cellobiose alludes to better tolerance to the same.

Afterward, to identify the regions of the proteins endowed with higher flexibility, the root-mean-square fluctuation (RMSF) of the protein backbone was calculated for the proteins with and without a ligand along the MD trajectories. The RMSF as a function of residue number of the ligand-bound proteins is shown in Additional file 1: Figure S8, and the RMSF-colored snapshots from multiple conformations of the protein are shown in Fig. 4b, d. Comparing the protein backbone RMSF of the ligand-bound TrCBH1 to PfCBH1, we see both proteins exhibit increased fluctuation primarily in loops B1, A1, and A4. We noticed small fluctuations in the region between loops B3 and B4. Loops B1 and A1 are the substrate entrance loops, loop A4 is associated with product exit, while loops B3 is the exo-loop and it influences the substrate accessibility to the active site, B4 is the end of the active site tunnel, beyond the reducing end of the cellulose chain.

Overall, PfCBH1 shows a greater degree of flexibility in the substrate entrance loop (loop B1) and the loops adjoining the active sites (loops B2, B3, and A3). This suggests a more dynamic tunnel entrance in PfCBH1 as compared to that in TrCBH1. A more open substrate-binding site also seems to be positively correlated with the decreased substrate affinity we had earlier observed and possible increased endo-initiation activity of the enzyme. We noticed a high flexibility with the adjoining loop adjacent to loop B3 and B4 (identified as circle iii in Fig. 3), while it is not directly associated with the substrates its high flexibility suggests that it may be affecting product binding and expulsion. This stretch of amino acids natively missing in TrCBH1 may be contributing to the observed increased tolerance of PfCBH1 to cellobiose.

Similarly, the RMSF of the active site-bound cellononaose was calculated to examine how the ligand behaves on a per-binding-site basis in the CBH tunnels (Fig. 4c). We observed that PfCBH1 ligand exhibited a higher fluctuation across all subsites (−7 to −5 binding sites) when compared with TrCBH1. Consistent with our previous results, PfCBH1 ligand showed a relatively higher flexibility toward the entrance of the CBH active site (subsites −7/−6). This could be to compensate for the shorter PfCBH1 A1 loop. However, the more noticeable ligand fluctuation differences observed in PfCBH1 were in the substrate site around exo and product loops (−4 to +2) correlating with ‘the more open’ conformation observed with the loops enclosing this site (loops B2, B3, and A3).

**Fig. 5:**
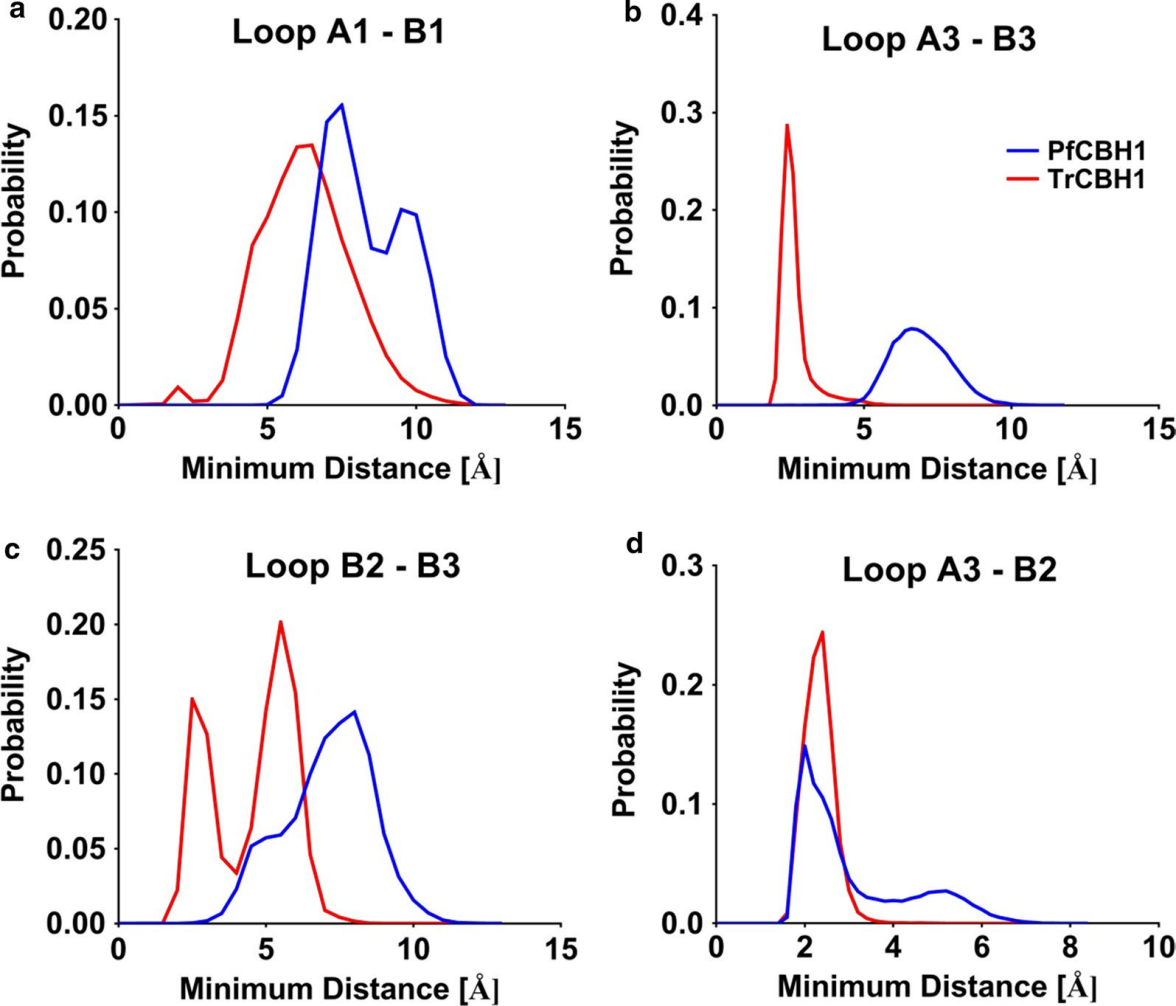
Histograms of the minimum distance between loops along cellulose binding paths. The minimum distance between loops A1 to B1 (**a**); loops A3 to B3 (**b**); loops B2 to B3 (**c**); and loops A3 to B2 (**d**) from 100-ns MD simulations of PfCBH1 and TrCBH1 are depicted. The distances have been measured in the presence of a ligand (bound to cellononaose)

Related to the RMSF analysis is the minimum distances between the loops during simulations. GH7 enzymes exhibit different loop–loop contacts [15]. To this end, the minimum distances between the putative tunnel entrance loops (A1–B1), and the loops across the tunnel’s active site—hypothesized to participate in endo/exo initiation events (A3, B2, and B3)—were estimated, and binned over 0.25 Å intervals (Fig. 5). Consistent with our previous observations, PfCBH1 demonstrated more open conformations in the entrance loops A1 and B1, with minimum distances of 7.5 and 9.5 Å as against a minimum average distance of 6.5 Å observed with TrCBH1. These open conformations facilitate the easier entrance of cellulosic substrates to the active site tunnel [3, 9, 15]. Similar trends were also observed with exo-loop (loop B3) interactions with its neighboring loops B2 and A3 (Fig. 5b, c). The exo-loop B3 interacts with adjacent loop A3 to form an enclosure over the catalytic site in the active site tunnel [3, 14, 31]. The unique ability of certain GH7 CBHs to conduct an endo-initiated attack of crystalline substrates is related to both the flexibility and the length of this loop, along with that of the nearby loop B2. Both of the loops (A3–B3) must open sufficiently to allow the entry into the active site of an internal part of a cellulose chain [14]. TrCBH1 maintains a minimum distance of about 3.5 Å over the course of the simulation consistent with previous report [15], whereas both PfCBH1 maintain a minimum distance of about 7 Å. In *P. chrysosporium* (PchCel7D), loops B3–A3 open as much as 12 Å [15].

In a similar manner, TrCBH1 exhibited two conformations—about 2.5 and 5.5 Å—of nearly equal probabilities between adjacent loops B2–B3, as against 8 Å exhibited by PfCBH1 in the same loops. We wish to state that we had earlier on pointed to a more open tunnel formed at the side walls of the ligand binding tunnel corresponding to this region in PfCBH1 when compared with TrCBH1. This current observation (minimum loop distance) offers a possible explanation for the phenomenon. Putting this in context, we can infer that PfCBH1 has a higher endo-initiation propensity on cellulose surface than TrCBH1 but at a lesser magnitude than PchCel7D and or known endoglucanases [3, 38]. On the other hand, we observed that PfCBH1 and TrCBH1 exhibit approximately the same dynamic range of distances between loops A3–B2 (Fig. 5d). This trend was equally reported with cellobiohydrolase from *Geotrichum candidum* [14]. The authors explained this behavior to mean a conservation of exo-initiated attack on crystalline cellulose substrates characteristic of GH7 CBH’s. It could be inferred that while exo-initiated activity remains the hallmark of GH7 CBH’s, nature has evolved variants with different functionality.

## Conclusions

In this study, we have explored the functional properties of a previously unexplored GH7 cellobiohydrolase from the hypercellulolytic fungus—*P. funiculosum* NCIM1228, and compared its saccharification potentials to that from *T. reesei* which is being widely used in the commercial cocktails. Our study indicates that PfCBH1 is superior to TrCBH1, considering properties such as specific activity, catalytic efficiency, and tolerance to inhibitors. Structural comparison with existing GH7 cellobiohydrolases confirms the conservation of essential amino acid residues characteristic of GH7 cellobiohydrolases. However, we observed and highlighted structural differences between PfCBH1 and TrCBH1 which tend to explain why PfCBH1 displayed higher enzyme functionalities compared to TrCBH1. Our further probe through molecular dynamic simulations confirmed the previously highlighted variations most especially in regions enclosing the catalytic pathway, indicating easier entrance of cellulosic substrates to the active site tunnel and higher tolerance level of PfCBH1 towards its product cellobiose. Building on the previous knowledge that the prospecting and engineering for higher activity represents one of the most important research activities pivotal to the commercialization of biofuel processes based on enzymatic depolymerization of polysaccharides, we thus present cellobiohydrolase 1 from *P. funiculosum* NCIM1228 as a viable alternative or possible replacement for cellobiohydrolase 1 from *T. reesei* in industrial cellulase cocktails.

## Methods

### Protein production and purification

*Penicillium funiculosum* (NCIM 1228) previously identified and characterized as a hypercellulolytic fungus [5] was maintained on potato dextrose agar and cultivated in a cellulase-inducing medium containing soya peptone (24 g/L), KH_2_PO_4_ (5.9 g/L), (NH4)_2_SO_4_ (3.12 g/L), CaCl_2_·2H_2_O (0.05 g/L), yeast extract (0.05 g/L), wheat bran (24 g/L) and Avicel (21.4 g/L); the final pH was adjusted to 5.5. The cellulase inducing medium in Erlenmeyer flasks was inoculated with four plugs (1 mm diameter) from the edge of the actively growing fungi, respectively. The flasks were kept at 30 °C for 6 days (optimal cellulase induction had been previously observed at this period of incubation) with orbital shaking at 150 rpm (Innova 44, Eppendorf AG, Germany). Induced cultures were centrifuged at 7000 rpm for 10 min at 4 °C; then, supernatants were filtered using syringe filters with a 0.45-μm PVDF membrane (Millipore, Germany).

The protein PfCBH1 was purified from the crude protein to homogeneity in three steps using a sequence of hydrophobic interaction chromatography (HIC), ion-exchange chromatography (IEC), and finally, a HIC of the flow through again, as previously described [22] with modifications. All separations were performed on an NGC™ Medium-Pressure Chromatography System (Bio-Rad, USA) at room temperature. Briefly, clarified crude protein previously conditioned with sodium acetate buffer (pH 5) containing 1 M (NH_4_)_2_SO_4_ was applied to a Phenyl Sepharose 6 FF High Sub column (GE Healthcare, USA) equilibrated with 50 mM sodium acetate buffer (pH 5), containing 1.0 M (NH_4_)_2_SO_4_. Proteins were eluted at 5 mL/min by the stepwise reduction of the (NH_4_)_2_SO_4_ concentration from 1 to 0 M and a stepwise increment in sodium acetate buffer from 0 to 80 percent over 1 column volume till a stable UV280 readout, and the second increment of 100% over 1 column volume. The obtained fractions were assessed for cellobiohydrolase activity.

Fractions active for cellobiohydrolase activity was pooled and dialyzed against 20 mM Tris–Cl buffer pH 7 on a G-25 fine Sephadex resin packed in an XK16/20 column (GE Healthcare, USA). Subsequently, the sample was applied to a Q-Sepharose Fast Flow resin packed in an XK16/20 column (GE Healthcare, USA), equilibrated in 20 mM Tris–HCl, pH 7, and proteins were eluted using a linear gradient 0–500 mM NaCl over 20 column volumes at 2.5 mL/min. To polish the purified protein, the fractions positive for cellobiohydrolase activity were pooled and dialyzed against 50 mM acetate buffer pH 5, containing 1 M (NH_4_)_2_SO_4_. The protein was applied to a Phenyl Sepharose 6 FF High Sub column (GE Healthcare, USA), and then eluted using an (NH_4_)_2_SO_4_ gradient (1 to 0 M over 120 min) at 1 mL/min. Fractions (2 mL) were collected, dialyzed against sodium acetate buffer 50 mM, pH 5, and then assayed for cellobiohydrolase activity. The obtained preparation was ascertained for its purity on SDS-PAGE [42], protein concentration was determined by the Bicinchoninic acid (BCA) method using bovine serum albumin as a standard [42].

### Antibody production and western blotting

To ascertain the veracity of the purified protein sample as cellobiohydrolase 1 (CBH1), we performed a Western blot analysis using anti-PfCBH1 antibody generated commercially by ABClonal (Wuhan, China) following standard procedures. In short, a “TYPTNATGTPGAARGTC” amino acid sequence between positions 391 and 407 of the cellobiohydrolase 1 polypeptide from *P. funiculosum* NCIM 1228 was synthesized and conjugated to the Keyhole Limpet Hemocyanin protein as a carrier. New Zealand white rabbits were immunized with the conjugated protein. The quality of purified anti-PfCBH1 antibody was assessed by ELISA using the pre-immune serum as a control.

For Western blotting, samples were heated at 95 °C for 10 min in a loading buffer, and equal amounts of protein were fractionated by sodium dodecyl sulfate-polyacrylamide gel (SDS-PAGE) electrophoresis and then transferred onto polyvinylidene difluoride (PVDF) membranes with a Trans-Blot Cell System (Bio-Rad) at 25 V for 20 min. The membrane blocking was done by incubating it with 3% BSA (made in 1X PBS) for one hour. Then, the blocked membrane was washed twice with PBST (1X PBS with 0.05% Tween-20) for 5 min each and finally with PBS. The blot was then incubated with anti-PfCBH1 antibody diluted 1:1000 in 3% BSA in PBS solution for 1 h at room temperature.

Again, the blot was washed (as mentioned above) and then incubated for an hour with horseradish peroxidase (HRP)-conjugated anti-rabbit secondary antibody (Sigma, USA) which had been diluted (1:2000) and prepared in the blocking solution. The blot was washed twice with PBST and once with PBS, and the color development was detected using 10 mL of the developing solution (1X PBS with 10 mg DAB tetrahydrochloride hydrate (Amresco, USA) and 30 μL of hydrogen peroxide).

### Differential scanning fluorimetry

The thermal stability of the purified protein as a function of variability in the environmental pH was evaluated by differential scanning fluorimetry. The assay was performed using a CFX96 Real-Time PCR System (Bio-Rad, USA). Briefly, a 5 µL of 25 × SYPRO orange dye (Invitrogen, USA) was added to 10 μL of protein at 1 mg/mL in 50 mM citrate phosphate buffer at different pH—2.4, 2.8, 3.2, 3.5, 3.8, 4.2, 4.6, 5.0, 5.5, 6.0, 6.4, and 7.1. The reactions were made up to 50 µL in a 96-well plate, and the samples were heated at 0.5 °C per 5 s from 25 to 95 °C. The fluorescence intensity (excitation/emission: 450 to 490 nm/560 to 580 nm) was measured every 0.5 °C. Thermal midpoint (*T*_m_) values of proteins were determined by the CFX Manager Program (Bio-Rad) based on a calculation of the negative first derivative [24]. Wells containing SYPRO orange dye with the different buffers served as a control to correct background fluorescence.

Experiments were carried out in triplicates and *T*_m_ values obtained were calculated for each well and compared to the control *T*_m_ values. The obtained *T*_m_ values were plotted against pH, and the optimal parameters obtained as amplitude and mean, respectively, by the robust fitting of experimental data to the Gaussian curve using GraphPad Prism version 7.00 for Windows, GraphPad Software, La Jolla California USA, http://www.graphpad.com.

### Measurement of PfCBH1 activity on cellobiohydrolase substrates

The activities of the purified enzyme towards cellobiohydrolase substrates—microcrystalline cellulose (Avicel PH-101), and chromogenic substrates—*p*-nitrophenyl-β-d-cellobioside (*p*NPC) and from *p*-nitrophenyl-β-d-lactopyranoside (*p*NPL) all obtained from Sigma, USA were measured as described earlier [5]. All experiments were conducted in 96 wells deep well plates. For enzyme activity on Avicel, 15 µg of purified enzyme in 30 µL was mixed with 100 µL of substrates at 1% Avicel in sodium acetate buffer pH 5 and incubated for 1 h at 50 °C. The reaction was terminated by the addition of DNSA reagent [43] and boiled for 10 min. The absorbance at 540 nm was measured relative to a glucose standard curve. One unit of enzyme activity was defined as the amount of protein that released 1 µmol of reducing sugar per min.

Activities on *p*NPL and *p*NPC were assayed by monitoring the release of *p*-nitrophenol. Briefly, 30 µL of purified enzyme dilutions was mixed with 100 µL of substrate (1 mM) and incubated for 20 min. The reaction was stopped by adding 130 µL of 1 M sodium carbonate (pH 11.5), and the release of 4-nitrophenol was quantified at 410 nm using a 4-nitrophenol standard curve. One unit of enzyme activity was defined as the amount of protein that released 1 µmol of *p*-nitrophenol per min.

### Determination of pH and temperature optima of PfCBH1

For the evaluation of temperature and pH interplay on PfCBH1 activity, the activity on Avicel was tested as above using pH conditions described in “Differential Scanning Fluorimetry” section but the samples were incubated at temperatures 40 to 70 °C step 10°, and protein load was 0.5 mg/mL. Experiments were carried out in triplicates. Obtained data were smoothed with Savistsky–Golay smoothing [44] to reduce short-term fluctuations and highlight global trends.

### Enzyme kinetics and cellobiose inhibition of PfCBH1

The kinetic parameters of PfCBH1 were determined in *p*NPL solutions of 0.0, 0.2, 0.4, 0.8, 1.6, 3.2, 4.8, 6.4, and 8.0 mM. *p*NPL was incubated with 1 µM PfCBH1 at 50 °C in 50 mM sodium acetate buffer, pH 4.4 as described in “Measurement of PfCBH1 activity on cellobiohydrolase substrates” section. Inhibition experiments were equally carried out under the same conditions above in the presence of 1000 µM cellobiose. Michaelis–Menten plots were constructed comparing the reaction rates obtained at different substrate concentrations in the absence and presence of inhibitor. Enzyme kinetics data were fit to the Michaelis–Menten expression by graphing reaction rates and substrate concentration values, to yield values for the apparent kinetic constants *V*_max_, *K*_m_, *k*_cat_, *k*_cat_/*K*_m_, and *K*_i_. Fitting and plots were generated using GraphPad Prism version 7.00 for Windows, GraphPad Software, La Jolla California USA, http://www.graphpad.com. All reactions were carried out in triplicates.

To determine the IC_50_ value (50% inhibition concentration) of PfCBH1 in the presence cellobiose, hydrolysis of 1 mM *p*NPL in the presence of different concentrations (0, 50, 100, 200, 400, 500, 1000, 2000, 4000, and 8000 µM) of cellobiose was performed as described in “Measurement of PfCBH1 activity on cellobiohydrolase substrates” section. Afterward, the data were analyzed using a four-parameter logistic equation in GraphPad Prism version 7.00 for Windows and the IC_50_ value determined.

### Oligomers profiling of PfCBH1

To investigate the nature of the products generated by PfCBH1 acting on cellodextrins, we conducted oligomers hydrolysis using cellobiose (G2), cellotriose (G3), cellotetraose (G4), cellopentaose (G5), and cellohexaose (G6). A 5 µM concentration of PfCBH1 was incubated with solutions of these compounds (500 µM) in 50 mM sodium acetate buffer (pH 4.4) for 30 min at 50 °C. Enzymes were inactivated by boiling at 95 °C for 5 min and were filtered through a 0.22 µm-pore-size syringe filter into glass high-performance liquid chromatography (HPLC) vials.

The hydrolysis products profiles and concentrations were assayed on a high-performance liquid chromatography system (Agilent Technologies, USA) equipped with Aminex HPX-87H anion exchange column (Bio-Rad, USA) and a refractive index detector. The filtered mobile phase (4 mM H_2_SO_4_) was used at a constant rate of 0.3 Lmin with column and RI detector temperatures maintained at 35 °C. Standards of glucose and oligomers at 1 g/L were separated using similar condition and areas obtained were used to calculate product concentration in the test samples.

### Comparative hydrolysis of microcrystalline cellulose (Avicel) by PfCBH1 and TrCBH1

The hydrolysis reaction mixtures contained 1% wt/vol microcrystalline cellulose—(Avicel PH-101; Sigma, USA) and 5 µM PfCBH1 in 50 mM sodium acetate buffer pH 4.8. The reactions were stopped at 1 and 24 h, respectively. Samples were filtered through a 0.22 µm-pore-size syringe filter into glass high-performance liquid chromatography (HPLC) vials. Glucose and cellobiose concentration estimated as described above.

To rate the efficiency of PfCBH1, products profiles and concentrations obtained were compared with that obtained from an equivalent load of CBH1 from *Hypocrea jecorina* (TrCBH1) procured from Sigma, USA—product number E641. The reactions were set up in duplicates, and data equally obtained in duplicates for each set on the HPLC system. Control setups containing substrates without enzymes at the different time points were used to rule out background Avicel hydrolysis.

### Comparative hydrolysis of pretreated wheat straws by PfCBH1 and TrCBH1

To evaluate the performance of purified PfCBH1 as against pure TrCBH1 (Sigma, USA) on pretreated biomass, a comparative hydrolysis of wheat straws that had been subjected to sodium hydroxide and ammonium hydroxide pretreatment (kindly provided by Prof. Arvind Lali) were used as substrates for the hydrolysis experiments in the presence of other core cellulolytic enzymes–endoglucanase (*ENDO5*, Lot 30702a), endoglucanase (*ENDO7*, Lot 111101a), betaglucosidase (*BGL*, Lot 141001), endo-xylanase (*BXYL*, Lot 101001d), and cellobiohydrolase II (*CBHII*, Lot 150501a) all procured from Megazyme, Ireland.

Design-expert software (Version 10; Stat-Ease, Inc., Minneapolis, MN; http://www.statease.com) was used to create the simplex-lattice designs and to analyze responses. An augmented quadratic design was implemented. The simplex-lattice design containing seven components required 41 runs for each of the pretreated wheat straws. A minimum enzyme proportion of 5% was used as a lower limit for TrCBH1, BGL, and CBHII. All pipetting mix were performed on Tecan Freedom Evo-2 150 Liquid Handler Automated workstation (Tecan Group Ltd., Switzerland). On completion of the dispensing program, the plates were sealed with adhesive PCR Plate Seals (Genetix, India) to prevent evaporation and biomass hydrolysis was carried out as previously reported [5].

Briefly, the reaction setup included pretreated wheat straws (previously graded through a 0.5 mm mesh and appropriately weighed out into 1.2 mL capacity 96-wells deep-well plates) at 5% w/v in a 250 µL final reaction volume containing the appropriate enzyme mix/ratio (total protein load of 2.5 mg/g dry biomass) in 50 mM sodium acetate buffer (pH 5.0). Enzymatic hydrolysis was performed at 50°C, shaking at 150 rpm (Innova 44, Eppendorf AG, Germany) for 30 h. Control experiments were carried out under the same conditions using substrates without enzymes (enzyme blank) and enzymes without substrates (substrate blank).

Following completion of hydrolysis, the plates were then centrifuged at 3000*g* for 10 min in a swinging bucket centrifuge (Eppendorf, Germany) to separate the solid residue from the digested biomass. The concentration of glucose in the hydrolysates was estimated using the glucose oxidase/peroxidase; GOPOD kit (Megazyme, Ireland) with d-glucose as a standard. All assays were carried out in triplicates and assayed twice (*n* = 6). Data were analyzed by ANOVA to develop a statistically based predictive model and the *F* ratio, *p* value, *R* square (*R* ^2^), adjusted *R* square (*R* ^2^), predicted *R* square (*R* ^2^), and adequate precision were calculated. Predicted ratios for optimal hydrolysis were validated, and the contributory effect of PfCBH1 and/or TrCBH1 in the presence of the optimal mix evaluated.

## 3D molecular modeling and illustration

The PDB coordinates of TrCBH1 with cellononaose chain (−7 to +2) were obtained from the theoretical model from PDB (PDB ID: 8CEL). The TrCBH1-cellononaose complex was used for homology modeling of PfCBH1 in the substrate-bound form using MODELLER v9.14 [45, 46]. Apo-PfCBH1 was obtained after removal of the cellononaose chain from the PfCBH1-cellononaose complex. The substrate + product complex (SP-PfCBH1 containing hydrolysed cellononaose chain, i.e., a celloheptaose (−7 to −1 subsites) and two glucose units cellobiose (+1 to +2 subsites) molecule) was obtained by removing the glycosidic bonds between the reducing-end cellobiose and the remainder of the cellononaose chain (between +1 and −1 subsites) in PfCBH1. The same procedure was followed for the TrCBH1-cellononaose chain.

Hence, six models were obtained by applying the methods mentioned above, two for each Apo, substrate, and substrate-product form in both PfCBH1 and TrCBH1 enzymes. The models were ranked according to the DOPE statistical potential score. Quality assessments including Ramachandran plots for the best model were performed with PROCHECK (http://services.mbi.ucla.edu/PROCHECK/). The structures were visualized using PyMOL molecular graphics system, version 1.4 (Schrödinger, New York, NY, USA).

### Molecular dynamics (MD) simulations

The PDB2PQR *Version* 2.1.1 [47] and PROPKA [48] was used to set working pH 4.5 for both enzymes activities by protonating HIS, ASP, and GLU. All MD simulations were performed using the Amber 14 package [49]. The topology and parameter files were generated by tleap, using the leaprcff99SB force-field for protein and the GLYCAM_06j-1 modified for carbohydrate molecules. The molecular systems were solvated with water molecules using the 10Ȧ pad of TIP3P water model. Neutralizing counter ions Na+ and Cl− species were added in respective systems. The energy minimization procedure is followed by heating and density equilibration methods; the minimization procedure includes 500 steps of Steep Descent (SD) algorithm with the protein fixed and carbohydrates free.

Following energy minimization, the systems were heated from 0 to 300 K over 50 ps with a collision frequency of 2.0 p/s, and weak harmonic restraints of 2 kcal/mol/Å^2^ on all atoms using Langevin thermostat for temperature regulation. Then, all the systems were subjected to short-time 50 ps runs at 300 K in the NPT ensemble with the 2 kcal/mol/Å^2^ weak restraints and pressure controlled using a Berendsen Barostat with a coupling constant of 1 ps and a target pressure of 1 bar. A final 5 ns of NPT ensemble was run at 300 K without harmonic restraints and a Langevin collision frequency of 2 p/s. The equilibrated density systems were then made for 100 ns duration production runs in the NVT ensemble at 300 K. The time step of 2 fs was used for all MD stages and all atoms involving hydrogen atoms were constrained using the SHAKE algorithm.

Long-range electrostatics were calculated in every stage using the Particle Mesh Ewald (PME) algorithm with a grid spacing of <1.0 Å. The non-bonded cut-off distance was set as 8 Å. All simulation coordinates were saved in single trajectory in all the six systems, and the trajectory was saved every 2 ps. The RMSD, RMSF analysis of trajectories was performed using CPPTRAJ module implemented in Amber 14. MM-GBSA [50] based end-point energy calculation was conducted to understand free energy differences between PfCBH1 and TrCBH1. The binding energies of PfCBH1 and TrCBH1 bound with cellononaose chain were calculated using 100 snapshots taken from 100 ns MD trajectory using the equation below:

1 $$\Delta G_{\text{bind}} = \, G_{\text{Complex}}\, - \, \left( { \, G_{\text{Protein}} \, - \, G_{\text{Carbohydrate}} } \right)$$

2 $$G \, = \, E_{\text{MM}} + \, G_{\text{sol}} \, {-} \, {\text{TS}}$$

3 $$E_{\text{MM}} = \, E_{\text{int}} + \, E_{\text{ele}} + \, E_{{{\text{vdw}} }}$$

4 $$G_{\text{sol}} = G_{\text{GB/PB}} + G_{\text{SA}},$$

where *E*_MM_ is the molecular mechanical energy; *E*_int_ is the internal energy (imparted by bonds, angles, and dihedrals); *E*_ele_ is the electrostatic energy; *E*_vdw_ is the van der Waals energy, and *TS* is the entropy contribution. *G*_sol_ is the solvation free energy, and it is made up of polar (*G*_GB/PB_) and nonpolar (*G*_SA_) energy components.

The nonpolar solvation energy (*G*_SA_) was calculated from the solvent-accessible surface area (SASA) using linear combination of pairwise orbitals (LCPO) method.

5 $$G_{\text{SA}} =\upgamma\cdot\text{ }SASA +\upbeta,$$

where surface tension coefficient γ and the offset β were the standard values of 0.00542/kcal/mol Å^2^ and 0.92/kcal/mol, respectively.

After considering all the equations for protein, carbohydrates and the complex, Eq. (1) can be reconstituted and expressed as

6 $$\Delta G_{\text{bind}} = \Delta E_{\text{MM}} + \, \Delta G_{\text{sol}} {-}T\Delta S,$$

where Δ*E*_MM_, Δ*G*_sol_, and *T*Δ*S* are the change in the mechanical energy, solvation energy and entropy between protein, ligand, and the complex. The solute entropy term (−*T*Δ*S*) was ignored in the present study as entropy differences are minuscule in such enzyme kinetic studies [51].

## Additional file

**Additional file 1: Table S1A.** Protein purification summary table for PfCBH1 using *p*NPC as substrate. **Table S1B.** Protein purification summary table for PfCBH1 using Avicel as substrate. **Table S2.** Compositional analysis of pretreated wheat straw used in the biomass hydrolysis experiments. **Table S3.** Enzymatic hydrolysis of ammonium hydroxide (AMM) and sodium hydroxide (ALK) pre-treated wheat straws by various mixtures of core cellulolytic enzymes. ** Table S4.** Numerical optimization of glucose release from pre-treated wheat straws. ** Figure S1.** Purification of *PfCBH1* from *P. funiculosum* NCIM 1228. Panel A represents the fractionation and purification scheme.; while panels B–D represent the chromatograms from Hydrophobic Interaction Chromatography 1 (HIC 1), Anion Exchange Chromatography (AEC) and a second Hydrophobic Interaction Chromatography HIC 2 respectively. **Figure S2.** *In vitro* thermal stability of purified cellobiohydrolase 1 from *T. reesei* under different pH conditions as determined by Differential Scanning Fluorimetry using SYPRO Orange. The T_m_ optimal and pH are reported as amplitudes and means of the Gaussian fittings respectively. **Figure S3.** Dose response of PfCBH1 to increasing concentration of cellobiose. The results are expressed as specific activity on *p*NPL and are the mean ± SEM (*n* = 3). **Figure S4.** Hydrolytic abilities of PfCBH1 singly and in tandem with ENDO5 on ALK (panel A) and AMM (panel B) pretreated wheat straw. On the *x*-axis, A represents PfCBH1, B represents ENDO5, C constitute a combination of A and B in ratio 50:50, while D constitute a combination of A and B in ratio 75:25. **Figure S5.** Multiple sequence alignment of PfCBH1 amino acid sequence with sequences of other GH7 CBHs. Sequences were retrieved from PDB and multiple sequence analysis performed using T-Coffee (http://tcoffee.crg.cat/). Each sequence is depicted with its PDB identifier and the pairwise percentage identity between PfCBH1 and each of the amino acid sequence indicated in parenthesis. The residues in red (EXDXXE motif) denote the catalytic residues, and the residues enclosed in red boxes represent the A1 to A4 and B1 to B4 loops enclosing the active-site tunnel. Loop nomenclature is made after Loop nomenclature is made after [3, 15]*. Talaromyces emersonii* (1Q9H), *Geotrichum candidum* (4ZZV), *Trichoderma harzianum* (2YOK), *Trichoderma reesei* (1CEL), *Aspergillus fumigatus* (4V1Z), *Phanerochaete chrysosporium* (1GPI), *Humicola grisea* (4CSI), *Dictyostelium purpureum* (4ZZP), *Heterobasidion annosum* (2XSP), *Dictyostelium discoideum* (4ZZQ), *Limnoria quadripunctata* (4GWA), *Daphnia pulex* (4XNN), and *Melanocarpus albomyces* (2RFW). **Figure S6.** Structural model of PfCBH1. Panel A, represents the best model of the PfCBH1 catalytic modules with a docked cellononaose ligand from the *T. reesei*—8CEL. The catalytic triad region is highlighted in purple, while the loops along the substrate binding path are colored red and labeled after [15]. Panel B is the Ramachandran plot validation of the modeled structure evaluated by PROCHECK. The Ramachandran statistics revealed that 90% of amino acid residues from the modeled structure were incorporated in the favored regions (A, B, and L) of the plot. 8.6% of the residues were in allowed regions (a, b, l, and p) of the plot. **Figure S7.** Comparison of the Root Mean Square Deviation (RMSD) over the 100 ns simulations of PfCBH1 and TrCBH1 in the absence of cellononaose bound ligand (Apo-PfCBH1 and Apo-TrCBH1), in the presence of cellononaose bound ligand (Substrate-PfCBH1 and Substrate-TrCBH1), and in the presence of celloheptaose with cellobiose (SP-PfCBH1 and SP-TrCBH1). **Figure S8.** Root-mean-square fluctuations (RMSF) of PfCBH1 and TrCBH1 complexed with cellononaose as a function of residue number. The regions corresponding to the loops are highlighted in green circles and labeled accordingly.

## Abbreviations

3D: three-dimensional
ALK: sodium hydroxide pretreated wheat straw
AMM: ammonium hydroxide pretreated wheat straw
ANOVA: analysis of variance
ASP: aspartic acid
BCA: bicinchoninic acid
BGL: betaglucosidase
BSA: Bovine serum albumin
BXYL: endo-xylanase
CBH1: cellobiohydrolase 1
CBHII: cellobiohydrolase II
CBM: carbohydrate-binding module
CBM1: carbohydrate-binding module 1
CD: catalytic domain
C-terminal: carboxy-terminus
Cα atoms: alpha carbon
DAB: diaminobenzidine
DNSA: 3,5-dinitrosalicylic acid
DOPE: discrete optimized protein energy
*E*_ele_: electrostatic energy
*E*_int_: internal energy
ELISA: enzyme-linked immunosorbent assay
*E*_MM_: molecular mechanic’s energy
ENDO5: endoglucanase glycoside hydrolase 5
ENDO7: endoglucanase glycoside hydrolase 7
*E*_vdw_: Vander Waals energy
G2: cellobiose
G3: cellotriose
G4: cellotetraose
G5: cellopentaose
G6: cellohexaose
*G*_GB/PB_: polar energy component
GH5: glycoside hydrolase 5
GH7: glycoside hydrolase 7
GLU: glutamic acid
GOPOD: glucose oxidase/peroxidase
*G*_SA_: nonpolar solvation energy
*G*_sol_: solvation free energy
HIC: hydrophobic interaction chromatography
HIS: histidine
HPLC: high-performance liquid chromatography
HRP: horseradish peroxidase
IC_50_: half maximal inhibitory concentration
IEC: ion-exchange chromatography
*k*_cat_: turnover number
*K*_i_: enzyme-inhibitor complex equilibrium dissociation constant
*K*_m_: michaelis constant
LCPO: linear combination of pairwise orbitals
MD: molecular dynamics simulations
MM-GBSA: molecular mechanics generalized Born surface area
NCIM: National Collection of Industrial Microorganisms
PBS: phosphate-buffered saline
PBST: phosphate-buffered saline/tween
PchCel7D: *Phanerochaete chrysosporium* cellobiohydrolase 7D
PCR: polymerase chain reaction
PDB: Protein Data Bank
PfCBH1: *Penicillium funiculosum* cellobiohydrolase 1
PME: Particle Mesh Ewald
*p*NPC: *p*-nitrophenyl-β-d-cellobioside
*p*NPL: *p*-nitrophenyl-β-d-lactopyranoside
PVDF: polyvinylidene difluoride
RI: Refractive Index
RMSD: root-mean-square deviation
RMSF: root-mean-square fluctuations
SASA: solvent-accessible surface area
SD: Steep Descent
SDS-PAGE: sodium dodecyl sulfate polyacrylamide gel electrophoresis
SP: substrate + product complex
*T*_m_: thermal midpoint
TrCBH1: *Trichoderma reesei* or *Hypocrea jecorina* cellobiohydrolase 1
UV: ultraviolet radiation
*V*_max_: maximum velocity
Δ*E*_MM_: change in the mechanical energy
Δ*G*: Gibbs free energy
Δ*G*_sol_: change in solvation energy
Δ*H*: enthalpy
Δ*S*: entropy
