# Supplementary material for: Comparative insights into the saccharification potentials of a relatively unexplored but robust Penicillium funiculosum glycoside hydrolase 7 cellobiohydrolase
Source: Biotechnol Biofuels. 2017 Mar 20;10:71. doi: 10.1186/s13068-017-0752-x (PMC5360062; doi:10.1186/s13068-017-0752-x)
Supplement: Supplementary file 1 — Additional file 1: Table S1A. Protein purification summary table for PfCBH1 using pNPC as substrate. Table S1B. Protein purification summary table for PfCBH1 using Avicel as substrate. Table S2. Compositional analysis of pretreated wheat straw used in the biomass hydrolysis experiments. Table S3. Enzymatic hydrolysis of ammonium hydroxide (AMM) and sodium hydroxide (ALK) pre-treated wheat straws by various mixtures of core cellulolytic enzymes. Table S4. Numerical optimization of glucose release from pre-treated wheat straws. Figure S1. Purification of PfCBH1 from P. funiculosum NCIM 1228. Panel A represents the fractionation and purification scheme.; while panels B–D represent the chromatograms from Hydrophobic Interaction Chromatography 1 (HIC 1), Anion Exchange Chromatography (AEC) and a second Hydrophobic Interaction Chromatography HIC 2 respectively. Figure S2. In vitro thermal stability of purified cellobiohydrolase 1 from T. reesei under different pH conditions as determined by Differential Scanning Fluorimetry using SYPRO Orange. The Tm optimal and pH are reported as amplitudes and means of the Gaussian fittings respectively. Figure S3. Dose response of PfCBH1 to increasing concentration of cellobiose. The results are expressed as specific activity on pNPL and are the mean ± SEM (n = 3). Figure S4. Hydrolytic abilities of PfCBH1 singly and in tandem with ENDO5 on ALK (panel A) and AMM (panel B) pretreated wheat straw. On the x-axis, A represents PfCBH1, B represents ENDO5, C constitute a combination of A and B in ratio 50:50, while D constitute a combination of A and B in ratio 75:25. Figure S5. Multiple sequence alignment of PfCBH1 amino acid sequence with sequences of other GH7 CBHs. Sequences were retrieved from PDB and multiple sequence analysis performed using T-Coffee (http://tcoffee.crg.cat/). Each sequence is depicted with its PDB identifier and the pairwise percentage identity between PfCBH1 and each of the amino acid sequence indicated in parenthes [file 13068_2017_752_MOESM1_ESM.docx]

**Supplementary Table 1A.** Protein purification summary table for PfCBH1 using pNPC as substrate

| Step | Fraction | Volume (ml) | Protein conc (mg/ml) | Activity (IU/ml) | Total Activity (units*ml) | Specific Activity (units/mg) | Fold Purification | % Yield |
| --- | --- | --- | --- | --- | --- | --- | --- | --- |
| 1 | Crude | 200 | 5.6 | 0.29 | 58 | 0.05 | 1.0 | 100 |
| 2 | HIC pool | 60 | 2.8 | 0.15 | 9 | 0.05 | 1.0 | 16 |
| 3 | GPC desalted | 120 | 1.0 | 0.04 | 5 | 0.04 | 0.8 | 9 |
| 4 | AEC flow through | 50 | 0.8 | 0.11 | 5 | 0.14 | 2.8 | 9 |
| 5 | HIC 2 pool (CBH fractions) | 30 | 1.4 | 0.12 | 4 | 0.09 | 1.8 | 7 |

**Supplementary Table 1B.** Protein purification summary table for PfCBH1 using Avicel as substrate

| Step | Fraction | Volume (ml) | Protein conc (mg/ml) | Activity (IU/ml) | Total Activity (units*ml) | Specific Activity (units/mg) | Fold Purification | % Yield |
| --- | --- | --- | --- | --- | --- | --- | --- | --- |
| 1 | Crude | 200 | 5.6 | 1.48 | 296 | 0.26 | 1.0 | 100 |
| 2 | HIC pool | 60 | 2.8 | 0.20 | 12 | 0.07 | 0.3 | 4 |
| 3 | GPC desalted | 120 | 1.0 | 0.18 | 22 | 0.18 | 0.7 | 7 |
| 4 | AEC flow through | 50 | 0.8 | 0.14 | 7 | 0.18 | 0.7 | 2 |
| 5 | HIC 2 pool (CBH fractions) | 30 | 1.4 | 0.20 | 6 | 0.14 | 0.5 | 2 |

**Supplementary Table 2.** Compositional analysis of pretreated wheat straw used in the biomass hydrolysis experiments as per NREL protocol

| Chemical Composition | % Distribution | |
| --- | --- | --- |
|  | ALK | AMM |
| Glucose | 88.0 | 68.3 |
| Xylose | 5.1 | 11.1 |
| Arabinose | 0.5 | 2.5 |
| Lignin | 5.7 | 15.1 |
| Ash | 0.8 | 3.1 |
| Crystallinity Index (per gram of cellulose) | 0.99 | 1.30 |

**Supplementary Table 3.** Enzymatic hydrolysis of ammonium hydroxide (AMM) and sodium hydroxide (ALK) pre-treated wheat straws by various mixtures of core cellulolytic enzymes***.

| Std | A:  TrCBH1  (GH7)  % | B: BGL  (GH3)  % | C:  BXYL  GH11)  % | D:  ENDO  GH5)  % | E:  ENDO  (GH7)  % | F:  PfCBH1  (GH7) % | G:  CBHII  (GH6)  % | Sum |  | **AMM**  **glucan hydrolysis**  **(%)** | | **ALK**  **glucan hydrolysis (%)** | |
| --- | --- | --- | --- | --- | --- | --- | --- | --- | --- | --- | --- | --- | --- |
|  |  |  |  |  |  |  |  |  |  | **Mean** | **SE** | **Mean** | **SE** |
| 1 | 90.0 | 5.0 | 0.0 | 0.0 | 0.0 | 0.0 | 5.0 | 100.0 |  | **5.35** | **0.11** | **7.92** | **0.23** |
| 2 | 5.0 | 90.0 | 0.0 | 0.0 | 0.0 | 0.0 | 5.0 | 100.0 |  | **1.65** | **0.06** | **2.04** | **0.07** |
| 3 | 5.0 | 5.0 | 85.0 | 0.0 | 0.0 | 0.0 | 5.0 | 100.0 |  | **2.11** | **0.07** | **1.84** | **0.35** |
| 4 | 5.0 | 5.0 | 0.0 | 85.0 | 0.0 | 0.0 | 5.0 | 100.0 |  | **4.83** | **0.08** | **12.43** | **0.57** |
| 5 | 5.0 | 5.0 | 0.0 | 0.0 | 85.0 | 0.0 | 5.0 | 100.0 |  | **5.03** | **0.15** | **12.52** | **0.34** |
| 6 | 5.0 | 5.0 | 0.0 | 0.0 | 0.0 | 85.0 | 5.0 | 100.0 |  | **9.10** | **0.72** | **13.17** | **0.45** |
| 7 | 5.0 | 5.0 | 0.0 | 0.0 | 0.0 | 0.0 | 90.0 | 100.0 |  | **2.27** | **0.05** | **3.86** | **0.13** |
| 8 | 47.5 | 47.5 | 0.0 | 0.0 | 0.0 | 0.0 | 5.0 | 100.0 |  | **3.97** | **0.11** | **5.48** | **0.23** |
| 9 | 47.5 | 5.0 | 42.5 | 0.0 | 0.0 | 0.0 | 5.0 | 100.0 |  | **4.18** | **0.08** | **6.90** | **0.35** |
| 10 | 47.5 | 5.0 | 0.0 | 42.5 | 0.0 | 0.0 | 5.0 | 100.0 |  | **9.63** | **0.24** | **20.62** | **0.48** |
| 11 | 47.5 | 5.0 | 0.0 | 0.0 | 42.5 | 0.0 | 5.0 | 100.0 |  | **9.16** | **0.93** | **17.81** | **0.31** |
| 12 | 47.5 | 5.0 | 0.0 | 0.0 | 0.0 | 42.5 | 5.0 | 100.0 |  | **7.52** | **0.34** | **13.90** | **0.30** |
| 13 | 47.5 | 5.0 | 0.0 | 0.0 | 0.0 | 0.0 | 47.5 | 100.0 |  | **4.34** | **0.05** | **6.42** | **0.46** |
| 14 | 5.0 | 47.5 | 42.5 | 0.0 | 0.0 | 0.0 | 5.0 | 100.0 |  | **1.77** | **0.05** | **2.28** | **0.05** |
| 15 | 5.0 | 47.5 | 0.0 | 42.5 | 0.0 | 0.0 | 5.0 | 100.0 |  | **2.93** | **0.07** | **8.76** | **0.24** |
| 16 | 5.0 | 47.5 | 0.0 | 0.0 | 42.5 | 0.0 | 5.0 | 100.0 |  | **3.21** | **0.08** | **9.08** | **0.59** |
| 17 | 5.0 | 47.5 | 0.0 | 0.0 | 0.0 | 42.5 | 5.0 | 100.0 |  | **5.07** | **0.10** | **7.83** | **0.28** |
| 18 | 5.0 | 47.5 | 0.0 | 0.0 | 0.0 | 0.0 | 47.5 | 100.0 |  | **1.85** | **0.03** | **3.10** | **0.18** |
| 19 | 5.0 | 5.0 | 42.5 | 42.5 | 0.0 | 0.0 | 5.0 | 100.0 |  | **3.06** | **0.38** | **11.25** | **0.58** |
| 20 | 5.0 | 5.0 | 42.5 | 0.0 | 42.5 | 0.0 | 5.0 | 100.0 |  | **2.74** | **0.34** | **6.21** | **1.53** |
| 21 | 5.0 | 5.0 | 42.5 | 0.0 | 0.0 | 42.5 | 5.0 | 100.0 |  | **7.77** | **0.24** | **10.26** | **0.40** |
| 22 | 5.0 | 5.0 | 42.5 | 0.0 | 0.0 | 0.0 | 47.5 | 100.0 |  | **1.81** | **0.22** | **2.96** | **0.21** |
| 23 | 5.0 | 5.0 | 0.0 | 42.5 | 42.5 | 0.0 | 5.0 | 100.0 |  | **5.52** | **0.09** | **14.85** | **0.62** |
| 24 | 5.0 | 5.0 | 0.0 | 42.5 | 0.0 | 42.5 | 5.0 | 100.0 |  | **13.90** | **0.35** | **19.63** | **0.75** |
| 25 | 5.0 | 5.0 | 0.0 | 42.5 | 0.0 | 0.0 | 47.5 | 100.0 |  | **3.62** | **0.08** | **10.86** | **0.22** |
| 26 | 5.0 | 5.0 | 0.0 | 0.0 | 42.5 | 42.5 | 5.0 | 100.0 |  | **11.42** | **0.35** | **19.08** | **0.31** |
| 27 | 5.0 | 5.0 | 0.0 | 0.0 | 42.5 | 0.0 | 47.5 | 100.0 |  | **3.62** | **0.07** | **11.33** | **0.39** |
| 28 | 5.0 | 5.0 | 0.0 | 0.0 | 0.0 | 42.5 | 47.5 | 100.0 |  | **7.28** | **0.13** | **9.56** | **0.26** |
| 29 | 53.6 | 11.1 | 6.1 | 6.1 | 6.1 | 6.1 | 11.1 | 100.0 |  | **10.03** | **0.18** | **17.81** | **1.11** |
| 30 | 11.1 | 53.6 | 6.1 | 6.1 | 6.1 | 6.1 | 11.1 | 100.0 |  | **4.72** | **0.12** | **10.58** | **0.50** |
| 31 | 11.1 | 11.1 | 48.6 | 6.1 | 6.1 | 6.1 | 11.1 | 100.0 |  | **5.52** | **0.52** | **13.63** | **0.45** |
| 32 | 11.1 | 11.1 | 6.1 | 48.6 | 6.1 | 6.1 | 11.1 | 100.0 |  | **8.74** | **0.19** | **16.80** | **0.68** |
| 33 | 11.1 | 11.1 | 6.1 | 6.1 | 48.6 | 6.1 | 11.1 | 100.0 |  | **8.08** | **0.19** | **17.52** | **0.53** |
| 34 | 11.1 | 11.1 | 6.1 | 6.1 | 6.1 | 48.6 | 11.1 | 100.0 |  | **11.32** | **0.23** | **19.35** | **0.38** |
| 35 | 11.1 | 11.1 | 6.1 | 6.1 | 6.1 | 6.1 | 53.6 | 100.0 |  | **6.36** | **0.11** | **14.15** | **0.47** |
| 36 | 17.1 | 17.1 | 12.1 | 12.1 | 12.1 | 12.1 | 17.1 | 100.0 |  | **8.64** | **0.15** | **18.50** | **0.45** |
| 37 | 90.0 | 5.0 | 0.0 | 0.0 | 0.0 | 0.0 | 5.0 | 100.0 |  | **5.56** | **0.11** | **8.14** | **0.36** |
| 38 | 5.0 | 90.0 | 0.0 | 0.0 | 0.0 | 0.0 | 5.0 | 100.0 |  | **1.66** | **0.04** | **2.12** | **0.07** |
| 39 | 5.0 | 5.0 | 85.0 | 0.0 | 0.0 | 0.0 | 5.0 | 100.0 |  | **1.96** | **0.22** | **3.06** | **0.18** |
| 40 | 5.0 | 5.0 | 0.0 | 85.0 | 0.0 | 0.0 | 5.0 | 100.0 |  | **4.65** | **0.07** | **13.05** | **0.38** |
| 41 | 5.0 | 5.0 | 0.0 | 0.0 | 85.0 | 0.0 | 5.0 | 100.0 |  | **4.63** | **0.13** | **13.00** | **0.24** |

***Enzyme loadings are expressed as a proportion of the total protein loadings. The total protein loading for the experiments is 2.5mg/g dry matter. Hydrolysis was carried out at pH 5, 50^o^C, with 5% (w/v) pre-treated wheat straw. Percentage hydrolysis was calculated from the amount of glucose in the hydrolysate after 30 hours. Values were means with standard error of mean (SE) where n=6.

**Supplementary Table 4.** Numerical optimization of glucose release from pre-treated wheat straws

| **Constraints** | | | |  | **Solutions (%)** | |
| --- | --- | --- | --- | --- | --- | --- |
| Name | Goal | Lower Limit | Upper Limit |  | **For AMM^a^** | **For ALK^b^** |
| A: TrCBH1 (GH7) | is in range | 5 | 85 |  | 7 | 34 |
| B: BGL (GH3) | is in range | 5 | 90 |  | 5 | 5 |
| C: BXYL (GH11) | is in range | 0 | 85 |  | - | 11 |
| D: ENDO (GH5) | is in range | 0 | 85 |  | 35 | 27 |
| E: ENDO (GH7) | is in range | 0 | 85 |  | 10 | 4 |
| F: PfCBH1 (GH7) | is in range | 5 | 85 |  | 39 | 5 |
| G: CBHII (GH6) | is in range | 5 | 90 |  | 5 | 15 |
| Glucose | maximize |  |  |  | (17-19) | (23-25) |

^a^ AMM - ammonium hydroxide treated wheat straw

^b^ ALK - sodium hydroxide treated wheat straw

**SUPPLEMENTARY FIGURE LEGENDS**

**Supplementary Figure 1.** Purification of *PfCBH1* from *P. funiculosum* NCIM 1228. Panel A represents the fractionation and purification scheme.; while panels B – D represent the chromatograms from Hydrophobic Interaction Chromatography 1 (HIC 1), Anion Exchange Chromatography (AEC) and a second Hydrophobic Interaction Chromatography HIC 2 respectively.

**Supplementary Figure 2.** *In vitro* thermal stability of purified cellobiohydrolase 1 from *T. reesei* under different pH conditions as determined by Differential Scanning Fluorimetry using SYPRO Orange. The T_m_ optimal and pH are reported as amplitudes and means of the Gaussian fittings respectively.

**Supplementary Figure 3.** Dose response of PfCBH1 to increasing concentration of cellobiose. The results are expressed as specific activity on pNPL and are the means ± SEM (n = 3).

**Supplementary Figure 4.** Hydrolytic abilities of PfCBH1 singly and in tandem with ENDO5 on ALK (panel A) and AMM (panel B) pretreated wheat straw. On the x-axis, A represents PfCBH1, B represents ENDO5, C constitute a combination of A and B in ratio 50:50, while D constitute a combination of A and B in ratio 75:25.

**Supplementary Figure 5**. Multiple sequence alignment of PfCBH1 amino acid sequence with sequences of other GH7 CBHs. Sequences were retrieved from PDB and multiple sequence analysis performed using T-Coffee (http://tcoffee.crg.cat/). Each sequence is depicted with its PDB identifier and the pairwise percentage identity between PfCBH1 and each of the amino acid sequence indicated in parenthesis. The residues in red (EXDXXE motif) denote the catalytic residues, and the residues enclosed in red boxes represent the A1 to A4 and B1 to B4 loops enclosing the active-site tunnel. Loop nomenclature is made after Loop nomenclature is made after [1, 2]*. Talaromyces emersonii* (1Q9H), *Geotrichum candidum* (4ZZV), *Trichoderma harzianum* (2YOK), *Trichoderma reesei* (1CEL), *Aspergillus fumigatus* (4V1Z), *Phanerochaete chrysosporium* (1GPI), *Humicola grisea* (4CSI), *Dictyostelium purpureum* (4ZZP), *Heterobasidion annosum* (2XSP), *Dictyostelium discoideum* (4ZZQ), *Limnoria quadripunctata* (4GWA), *Daphnia pulex* (4XNN), and *Melanocarpus albomyces* (2RFW).

**Supplementary Figure 6**. Structural model of PfCBH1. Panel A, represents the best model of the PfCBH1 catalytic modules with a docked cellononaose ligand from the *T. reesei* - 8CEL. The catalytic triad region is highlighted in purple, while the loops along the substrate binding path are colored red and labeled after [1]. Panel B, is the Ramachandran plot validation of the modeled structure evaluated by PROCHECK. The Ramachandran statistics revealed that 90% of amino acid residues from the modeled structure were incorporated in the favored regions (A, B, and L) of the plot. 8.6% of the residues were in allowed regions (a, b, l, and p) of the plot.

S**upplementary Figure 7.** Comparison of the Root Mean Square Deviation (RMSD) over the 100 ns simulations of PfCBH1 and TrCBH1 in the absence of cellononaose bound ligand (Apo-PfCBH1 and Apo-TrCBH1), in the presence of cellononaose bound ligand (Substrate-PfCBH1 and Substrate-TrCBH1), and in the presence of celloheptaose with cellobiose (SP-PfCBH1 and SP-TrCBH1).

S**upplementary Figure 8.** Root-Mean-Square Fluctuations (RMSF) of PfCBH1 and TrCBH1 complexed with cellononaose as a function of residue number. The regions corresponding to the loops are highlighted in green circles and labeled accordingly.

**Supplementary Figure 1.**

**Supplementary Figure 2.**

**Supplementary Figure 3.**


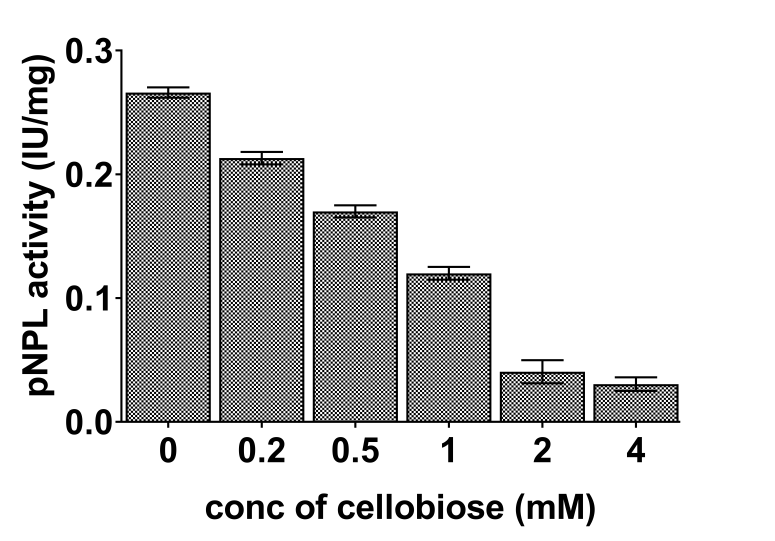


**Supplementary Figure 4.**


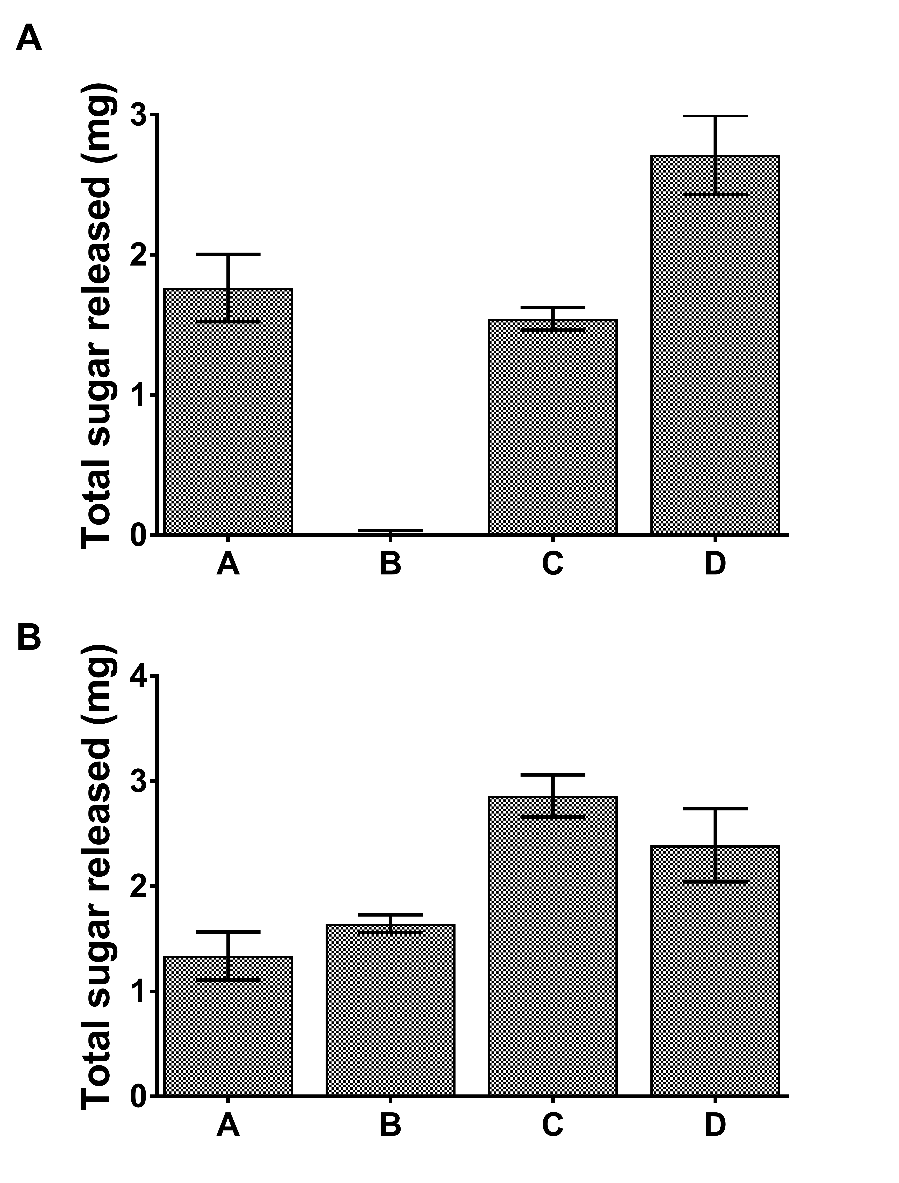


**Supplementary Figure 5.**


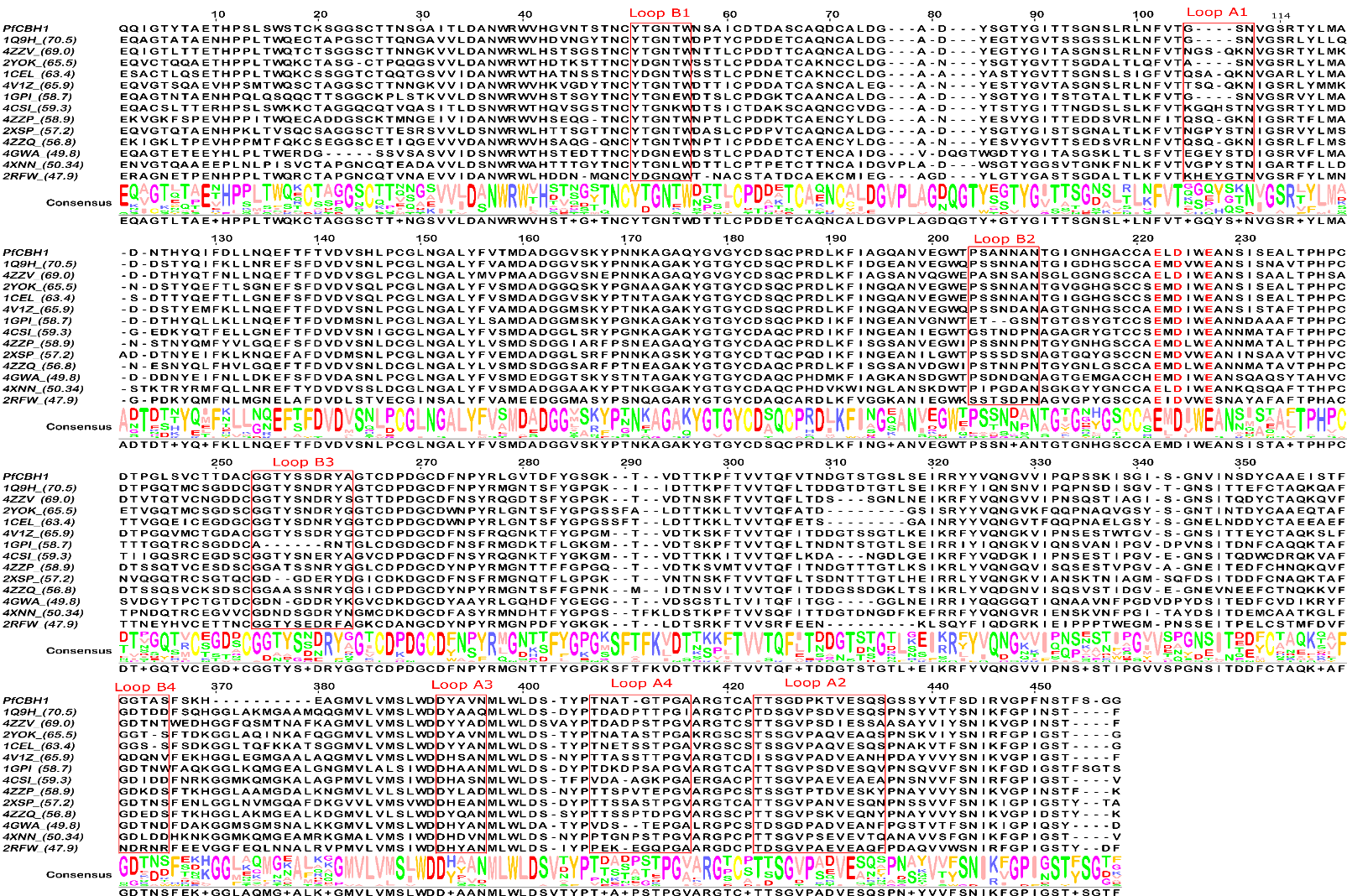


**Supplementary Figure 6.**


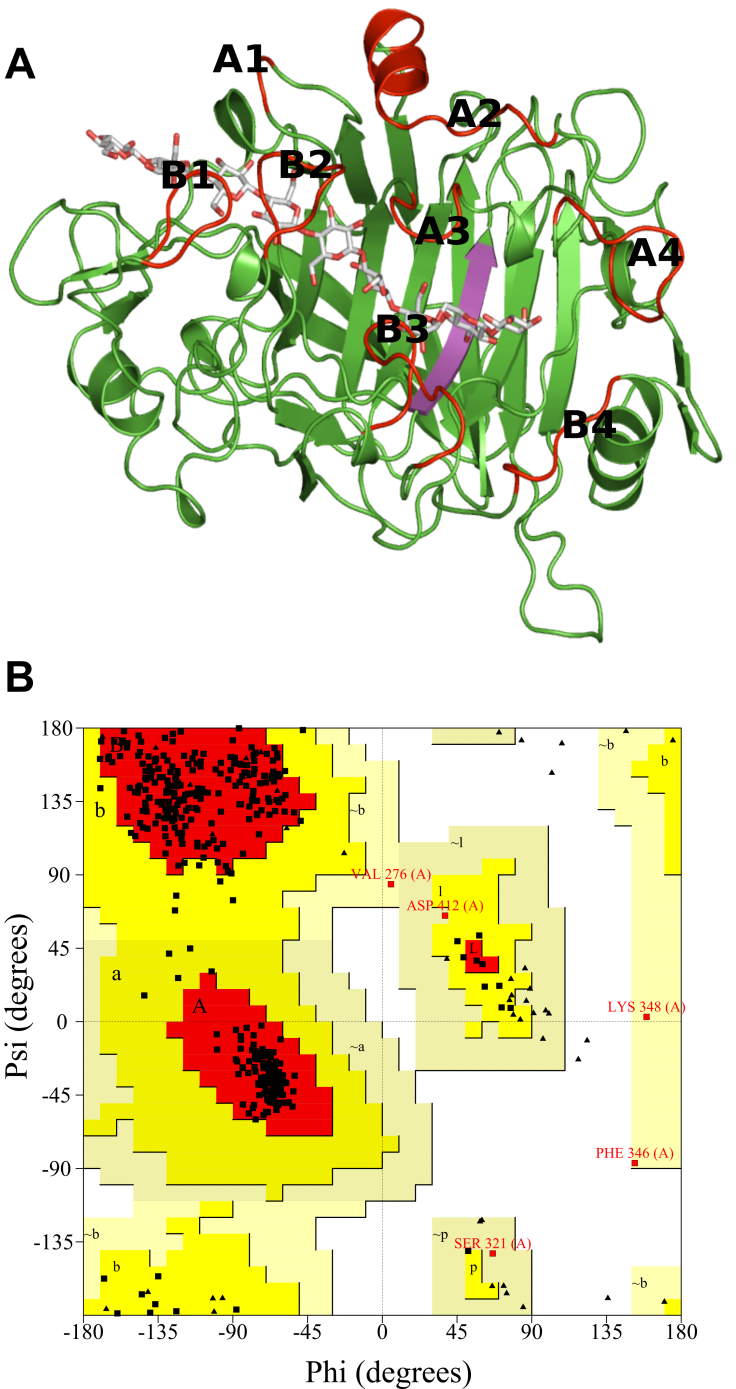


**Supplementary Figure 7.**


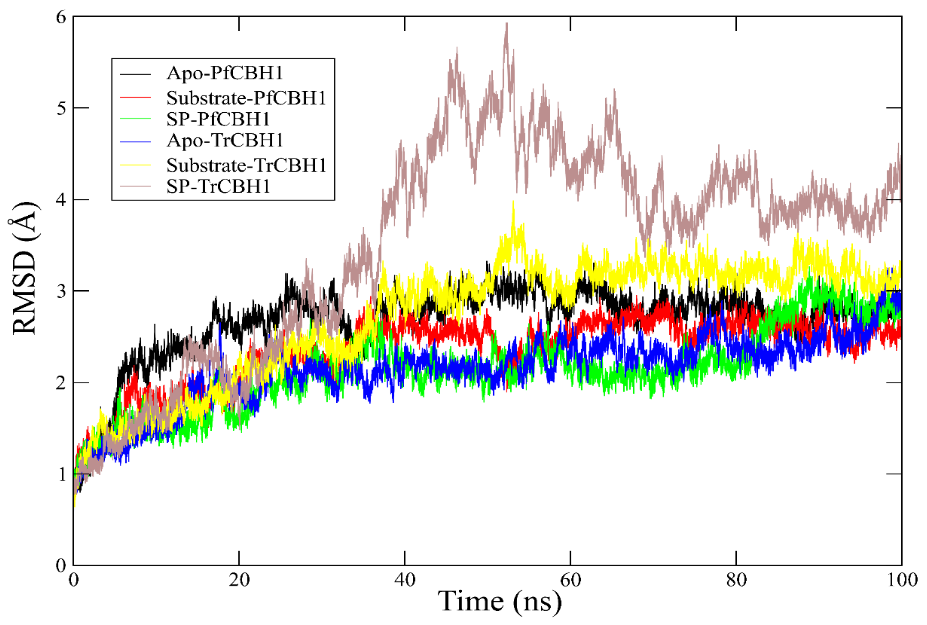


**Supplementary Figure 8.**

**
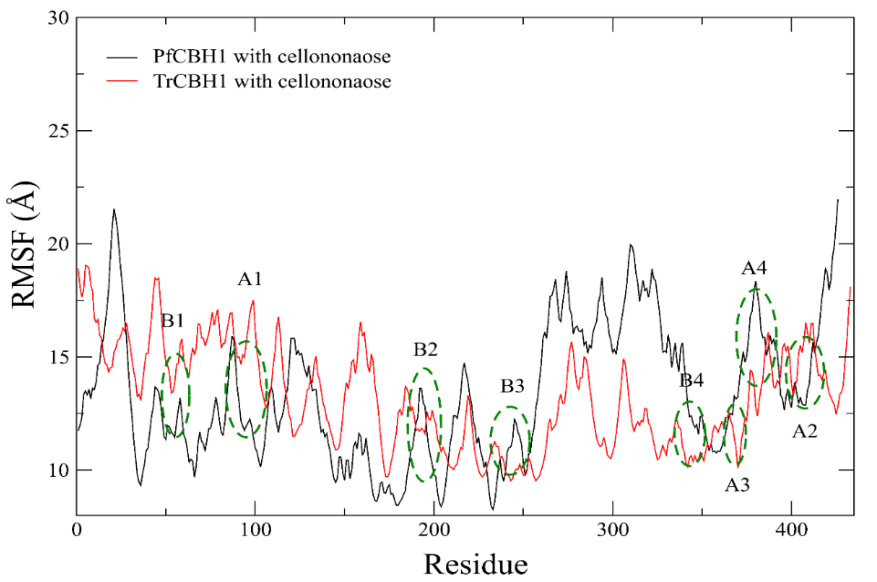
**

**REFERENCES**

1. Momeni MH, Payne CM, Hansson H, Mikkelsen NE, Svedberg J, Engstrom A, Sandgren M, Beckham GT, Stahlberg J: **Structural, biochemical, and computational characterization of the glycoside hydrolase family 7 cellobiohydrolase of the tree-killing fungus *Heterobasidion irregulare*.** *J Biol Chem* 2013, **288:**5861-5872.

2. Payne CM, Knott BC, Mayes HB, Hansson H, Himmel ME, Sandgren M, Stahlberg J, Beckham GT: **Fungal cellulases.** *Chem Rev* 2015, **115:**1308-1448.
